# Alpha-Gal Syndrome: Involvement of *Amblyomma americanum* α-D-Galactosidase and β-1,4 Galactosyltransferase Enzymes in α-Gal Metabolism

**DOI:** 10.3389/fcimb.2021.775371

**Published:** 2021-12-01

**Authors:** Surendra Raj Sharma, Gary Crispell, Ahmed Mohamed, Cameron Cox, Joshua Lange, Shailesh Choudhary, Scott P. Commins, Shahid Karim

**Affiliations:** ^1^ School of Biological, Environment and Earth Sciences, The University of Southern Mississippi, Hattiesburg, MS, United States; ^2^ Department of Medicine and Pediatrics, University of North Carolina, Chapel Hill, NC, United States; ^3^ Center for Molecular and Cellular Biosciences, The University of Southern Mississippi, Hattiesburg, MS, United States

**Keywords:** alpha-gal, tick, red meat allergy, alpha-gal syndrome, alpha-D-galactosidase, beta-1, 4 galactosyltransferase, N-glycan

## Abstract

Alpha-Gal Syndrome (AGS) is an IgE-mediated delayed-type hypersensitivity reaction to the oligosaccharide galactose-α-1, 3-galactose (α-gal) injected into humans from the lone-star tick (*Amblyomma americanum*) bite. Indeed, α-gal is discovered in salivary glands of lone-star tick; however, the tick’s specific intrinsic factors involved in endogenous α-gal production and presentation to host during hematophagy are poorly understood. This study aimed to investigate the functional role of two tick enzymes, α-D-galactosidase (ADGal) and β-1,4 galactosyltransferases (β-1,4GalT), in endogenous α-gal production, carbohydrate metabolism, and N-glycan profile in lone-star tick. The ADGal enzyme cleaves terminal α-galactose moieties from glycoproteins and glycolipids, whereas β-1,4GalT transfers α-galactose to a β1,4 terminal linkage acceptor sugars—GlcNAc, Glc, and Xyl—in various processes of glycoconjugate synthesis. An RNA interference approach was utilized to silence ADGal and β-1,4GalT in *Am. americanum* to examine their function in α-gal metabolism in tick and AGS onset. Silencing of ADGal led to the significant downregulation of genes involved in galactose metabolism and transport in *Am. americanum*. Immunoblot and N-glycan analysis of the *Am. americanum* salivary glands showed a significant reduction in α-gal levels in silenced tissues. However, there was no significant difference in the level of α-gal in β-1,4GalT-silenced tick salivary glands. A basophil-activation test showed a decrease in the frequency of activated basophil by ADGal-silenced salivary glands. These results provide an insight into the roles of ADGal and β-1,4GalT in α-gal production and presentation in ticks and the probable involvement in the onset of AGS.

## Introduction

Lone-star ticks (*Amblyomma americanum*) are highly competent arthropod vectors, which transmit a wide variety of viral and bacterial pathogens to mammals (Childs and Paddock, 2003; Goddard and Varela-Stokes, 2009; Sayler et al., 2014). The lone-star tick is associated with southern tick-associated rash illness (STARI) and Alpha-Gal Syndrome (AGS), a newly emerged delayed allergic reaction that occurs 3–6 h after eating beef, pork, or lamb (Commins et al., 2011; Commins and Platts-Mills, 2013). The development of specific IgE antibodies to the oligosaccharide galactose-α-1,3-galactose (α-gal) following the *Am. americanum* bites cause red-meat allergy (Van Nunen et al., 2009; Commins et al., 2011; Wuerdeman and Harrison, 2014; Crispell et al., 2019). Alpha-gal is found in the tissues of most mammals, including cattle, sheep, and swine but notably absent from humans and Great Apes. AGS is already common in several world regions; within 10 years, the US alone has confirmed a spike in cases from 12 in 2009 to >34,000 in 2019 (Binder et al., 2021); most strongly attributed to sensitization to α-gal is *Am. americanum* (Crispell et al., 2019). Several studies have reported the presence of α-gal in the midguts of *Ixodes ricinus*, salivary glands of *Haemaphysalis longicornis* and *Am. sculptum*, and both the salivary glands and saliva of *Ixodes scapularis* and *Am. americanum* (Hamsten et al., 2013a; Hamsten et al., 2013b; Araujo et al., 2016; Chinuki et al., 2016; Crispell et al., 2019; Choudhary et al., 2021).

AGS is most common in areas where the *Am. americanum* has been historically prevalent and expanded into the regions (e.g., Long Island, NY, known for the prevalence of *Ix. Scapularis*) (Monzón et al., 2016; Sonenshine, 2018). Range expansion of *Am. americanum* presents a significant public health threat in the northeastern US and beyond (Springer et al., 2015; Sonenshine, 2018; Raghavan et al., 2019) due to its established role in pathogen transmission and its link to AGS (Childs and Paddock, 2003; Monzón et al., 2016; Crispell et al., 2019; Commins, 2020; Sharma and Karim, 2021). The unexpected increase in AGS is a unique health concern because strict food allergen avoidance is the only way to prevent a life-threatening allergic reaction.

The IgE immune response associated with food allergies is classically directed against protein antigens; however, AGS is characterized by IgE that binds to the oligosaccharide epitope galactose-α-1,3 galactose (α-gal), a cross-reactive carbohydrate determinant (CCD), found explicitly in all non-primate mammals (Aalberse et al., 1981). The α-gal appears to be a common component of mammalian glycoconjugates such as glycolipids and glycoproteins. A large family of glycosyltransferases synthesizes these cellular glycoconjugates (Roseman, 2001; Hennet, 2002; Berg et al., 2014) found throughout the cells, tissues, and fluids of all lower mammals (Galili and Avila, 1999; Apostolovic et al., 2014; Takahashi et al., 2014; Hilger et al., 2016; Iweala et al., 2020). These glycosyltransferases catalyze the transfer of galactose through α1-2, α1-3, α1-4, α1-6, β1-3, and β1-4 linkage to diverse acceptor macromolecules (Hennet, 2002).

Our earlier combinatorial approach using N-glycome and proteome identified several unique and common α-gal antigens in salivary gland extracts and saliva of *Am. americanum* and *Ix. scapularis* (Crispell et al., 2019). We identified two tick enzymes, α-D-galactosidase (ADGal) and β-1,4-galactosyltransferase (β-1,4-GalT), and aimed to functionally characterize their role in α-gal expression, galactose metabolism, and n-glycan profile. When ticks feed on humans, they inject saliva antigens containing α-gal epitopes to the host, triggering the production of anti-α-Gal antibodies (Anti-Gal) (Commins and Platts-Mills, 2013). Thus, tick salivary antigens appear to be critical in the development of AGS (Crispell et al., 2019; Choudhary et al., 2021). During prolonged tick attachment on the host, when the tick secretes and delivers a salivary potion possibly containing α-gal-antigens to the host skin, it triggers or augments α-gal-directed IgE response (Araujo et al., 2016; Crispell et al., 2019; Choudhary et al., 2021). Surprisingly, continued exposure to ticks seems to augment the already existing IgE antibody response. However, it remains puzzling why the response is so strong and directed so consistently against the α-gal carbohydrate residue. In fact, the expression of α-gal is reported in various tick feeding stages of lone-star and the black-legged ticks (*Ixodes scapularis*). Yet, there is a fundamental gap in our knowledge of (a) how α-gal is produced or recycled within the salivary glands, (b) secreted in saliva to the host, and (c) which tick intrinsic factors are essential in α-gal production or expression in tick salivary gland during prolonged feeding on the host. The enzyme α1,3GalT, which synthesizes α-gal, remains unidentified in tick genomes (Sharma and Karim, 2021). Three variants of galactosyltransferase genes, i.e., β4galt-7; α4galt-1 and α4galt-2 from β-1,4-GalT, and α-1,4-GalT family, are shown to be involved in the α-gal synthesis pathway, and a piece of indirect evidence showed *Anaplasma phagocytophilum* infection in *Ixodes scapularis* cell culture induces an increase in expression of three other galactosyltransferases associated with increased levels of α-gal glycans (Cabezas-Cruz et al., 2018). A search for *Ix. scapularis* homologs in *Am. americanum* failed to yield any CDS in the existing NCBI transcriptome database. It is puzzling how the tick acquires and decorates its saliva antigens with α-gal and primes the host immune response. This study characterized the functional role of two tick enzymes, α-D-galactosidase (ADGal) and β-1,4-galactosyltransferase (β-1,4-GalT), in galactose metabolism of *Am. americanum*. Alpha-D-galactosidase (glycoside hydrolase/ADGal) catalyzes the breakdown of galactose from glycoproteins or glycolipids, and β-1,4-galactosyltransferase (β-1,4-GalT) is involved in the synthesis of Galβ1-4-GlcNac-disaccharide unit of glycoconjugates. Both enzymes are expressed during tick feeding progression, and we reveal the impact of each in overall α–gal expression in tick salivary glands in the context of alpha-gal syndrome.

## Materials and Methods

### Ethics Statement

All animal experiments were performed in strict accordance with the recommendations in the Guide for the Care and Use of Laboratory Animals of the National Institutes of Health, USA. The protocol for tick blood-feeding on sheep was approved by the Institutional Animal Care and Use Committee (IACUC) of the University of Southern Mississippi (protocol #15101501.2). All steps were taken to alleviate animal suffering.

### Materials

All common laboratory supplies and chemicals were procured through Bio-Rad (Hercules, CA, USA), Sigma-Aldrich (St. Louis, MO, USA), and Fisher Scientific (Grand Island, NY, USA) unless specifically noted.

### Ticks and Other Animals

Adult unfed lone-star ticks (*Amblyomma americanum*) were purchased from Oklahoma State University’s tick-rearing facility (Stillwater, OK, USA) and maintained at the University of Southern Mississippi following an established protocol (Patrick and Hair, 1975). Adult ticks were kept at room temperature at approximately 90% humidity with a photoperiod of 14 h of light and 10 h of darkness prior to infestation on sheep. The adult ticks were fed on sheep for time intervals between 1 and 11 days for tissue collection, depending on the experimental plan.

### DsRNA Synthesis and Tick Injections

The gene of interest was amplified using PCR with gene-specific primers and purified using the QIAquick PCR Purification Kit (QIAGEN, Germany). Gene-specific T7 promoter sequences were added to the 5’ and 3’ end of the purified product using PCR and were purified. The purified T7 PCR products were confirmed by sequencing and transcribed into dsRNA using the T7 Quick High Yield RNA Synthesis Kit (New England Biolabs, Ipswich, MA, USA). The dsRNA produced was purified *via* ethanol precipitation, and the concentration was measured using a nanodrop spectrophotometer and was analyzed on a 2% agarose gel. Unfed females were injected with 500 ng of the purified dsRNA using a 31-gauge needle and were maintained at 37˚C with 90% humidity for 24 h. The ticks were then fed on sheep. The ticks were removed at different time points to determine the expression (Bullard et al., 2016).

### Tick Tissue Dissection and Salivary Gland Extract

Partially fed female ticks removed from the sheep were dissected, and the salivary glands and midguts were removed and cleaned in ice-cold M199 buffer. Salivary glands and midguts from each time point were pooled together according to tissue type and stored in RNAlater (Life Technologies, Carlsbad, NM, USA) at −80°C until used (Bullard et al., 2016; Bullard et al., 2019). Tick salivary protein was extracted from partially blood-fed female *Am. americanum* following the method described previously (Crispell et al., 2019). The salivary protein extracts were stored immediately at −80°C until subsequent western blot analysis.

### RNA Isolation and cDNA Synthesis

Frozen tick tissues were placed on ice to thaw and followed by careful removal of RNAlater. RNA was isolated from the time point pooled salivary glands using Illustra RNAspin Mini kit (GE Healthcare Lifesciences) protocols. RNA concentration was measured using a nanodrop spectrophotometer and stored at −80°C or used immediately. Two µg of RNA was reverse transcribed using the iScript cDNA synthesis kit (Bio-Rad) to synthesize cDNA. The reverse transcription reaction was then heated in a Bio-Rad thermocycler under the following conditions: 5 min at 25°C, 30 min at 42°C, 5 min at 85°C, and hold at 10°C. The resultant cDNA was diluted to a working concentration of 25 ng/µl with nuclease-free water and stored at −20°C until used (Bullard et al., 2016).

### Quantitative Real-Time PCR

QRT-PCR was performed within the guidelines of Bio-Rad protocols provided with iTaq Universal SYBR Green Supermix. Briefly, 50 ng of cDNA was added to a 20 µl qRT-PCR reaction using SYBR Green supermix with 300 nM of each gene-specific primer. The samples were subjected to the following thermocycling conditions: 95°C for 30 s; 35 cycles of 95°C for 5 s and 60°C for 30 s with a fluorescence reading after each cycle; followed by a melt curve from 65°C to 95°C in 0.5°C increments. Each reaction was performed in triplicate along with no template controls (Bullard et al., 2016). Primers used for gene expression validation can be found in **Supplementary Table 2**. Gene expression validation was performed using β-actin and histone as the reference gene.

### Quantification of Total Bacterial Load

The total bacterial load in tick tissues was determined using the method described elsewhere (Narasimhan et al., 2014; Budachetri and Karim, 2015). Briefly, 25 µl volume reaction mixture contained 25 ng of tissue cDNA, 200 µM 16S RNA gene primer, and iTaq Universal SYBR Green Supermix (Bio-Rad) followed by a qPCR assay using following conditions: 94°C for 5 min followed by 35 cycles at 94°C for the 30 s, 60°C for 30 s and 72°C for 30 s. A standard curve was used to determine the copy number of each gene. The bacterial copy number was normalized against *Am. americanum* actin copy number in control tissues and gene-silenced tick, and each sample was run in triplicate.

### SDS-PAGE and Immunoblotting

SDS-Polyacrylamide Gel Electrophoresis and Immunoblotting were carried out using the methods described elsewhere (Crispell et al., 2019). Proteins extracted from the salivary glands (15 µg) were fractionated on a Mini-PROTEAN TGX Any kD, 4–20% gel (Bio-Rad) using SDS-PAGE. They were then transferred onto a nitrocellulose membrane in a Transblot cell (Bio-Rad). The transfer buffer consisted of 25 mM Tris-HCl and 192 mM glycine in 20% methanol. Blocking of non-specific protein binding sites was executed with 5% BSA in a TBS and Tween-20 solution. The membranes were incubated with α-galactose (M86) monoclonal IgM antibodies (Enzo Life Sciences, Farmingdale, NY, USA) at a dilution of 1:10 using an iBind western device (Life Technologies, Camarillo, CA, USA). The antigen-antibody complexes were visualized using a secondary horseradish peroxidase-conjugated goat anti-mouse IgM antibody (Sigma-Aldrich) at a dilution of 1:10,000. They were detected with SuperSignal chemiluminescent substrate (Pierce Biotechnology, Rockford, IL, USA) using a Bio-Rad ChemiDox XRS.

### Basophil Activation Assay With Tick Salivary Glands

Peripheral blood mononuclear cells (PBMCs) taken from a healthy, non-α-gal allergic donor (α-gal sIgE <0.10) were isolated using a Ficoll–Paque gradient (GE Healthcare, Chicago, IL, USA). Endogenous IgE was stripped from basophils within the PBMC fraction by incubating the cells with cold lactic acid buffer (13.4 mM lactic acid, 140 mM NaCl, 5 mM KCl) for 15 min. Basophils were sensitized with plasma from α-gal allergic and non-allergic subjects overnight in RPMI 1,640 cell culture media (Corning CellGro, Manassas, VA, USA) in the presence of IL-3 (1 ng/ml, RandD Systems, Minneapolis, MN, USA) at 37°C and 5% CO_2_. PBMCs were subsequently stimulated for 30 min with RPMI media, cetuximab (10 μg), rabbit anti-human IgE (1 μg; Bethyl Laboratories Inc., Montgomery, TX, USA), partially fed salivary gland extracts from *Am. americanum* (50 μg). Stimulation reactions were stopped with 20 mM EDTA, and PBMCs were incubated with a single panel of anti-human antibodies containing CD123-BV421 (BioLegend, San Diego, CA, USA), FITC lineage cocktail 1 (CD3, CD14, CD16, CD19, CD20, CD56, BD Biosciences, San Jose, CA, USA), HLA-DR-PerCP-Cy5.5, CD63-APC (eBiosciences, ThermoFisher, Waltham, MA, USA), and CD203c-PE (IOTest Beckman Coulter, Marseille, France) in flow cytometry staining buffer with 0.02% NaN_3_. Following two washes with PBS, samples were acquired on a CyAN™ ADP flow cytometer (Beckman Coulter, Brea, CA, USA) and analyzed using FlowJo v10 software (FlowJo LLC, Ashland, OR, USA). Cells were gated on an FSC *vs.* SSC dot plot, followed by FSC-H and FSC-A to eliminate doublets. Cells negative for lineage cocktail and HLA-DR were then gated on CD123, CD203c double-positive basophil and analyzed for the presence of CD63+ as a marker of basophil activation. Data analysis was performed using Prism version 7.03 (GraphPad Software, La Jolla, CA, USA). Mann–Whitney U-tests were used to compare the frequency of CD63+ basophils detected following stimulation with various compounds. A p-value < 0.05 was considered significant.

### N-Glycome Analysis of Tick Salivary Glands

N-linked glycans were released from 30 μl of *Am. americanum* salivary glands with an estimated protein concentration of 200 μg, after being reduced, alkylated, and then digested with trypsin in Tris-HCl buffer overnight. After protease digestion, the sample was passed through a C18 Sep-Pak cartridge, washed with 5% v/v acetic acid, and the glycopeptides were eluted with a blend of isopropanol in 5% v/v acetic acid before being dried by SpeedVac. The dried glycopeptide eluate was treated with a combination of PNGase A (Sigma) and PNGase F (New England Biolabs, Ipswitch, MA, USA) to release the N-linked glycans. The digest was then passed through a C18 Sep-Pak cartridge to recover the N-glycans. The N-linked glycans were then permethylated for structural characterization by mass spectrometry. Briefly, the dried eluate was dissolved with dimethyl sulfoxide and methylated with NaOH and methyl iodide. The reaction was quenched with water, and per-O-methylated carbohydrates were extracted with methylene chloride and dried under N2. The permethylated glycans were reconstituted in 1:1 MeOH:H_2_O containing 1 mM NaOH, then introduced to the mass spectrometer (Thermo Fusion Tribrid Orbitrap) with direct infusion at a flow rate of 0.5 μl/min. Full MS spectra and an automated “TopN” MS/MS program of the top 300 peaks were collected and fragmented with collision-induced fragmentation. These fragmentation data were used to confirm a Hex-Hex-HexNAc signature, both with a diagnostic fragment, as well as expected neutral losses.

## Results

### Temporal Expression of α-D-Galactosidase and β-1,4-Galactosyltransferase in Tick Salivary Glands

**Figure 1:**
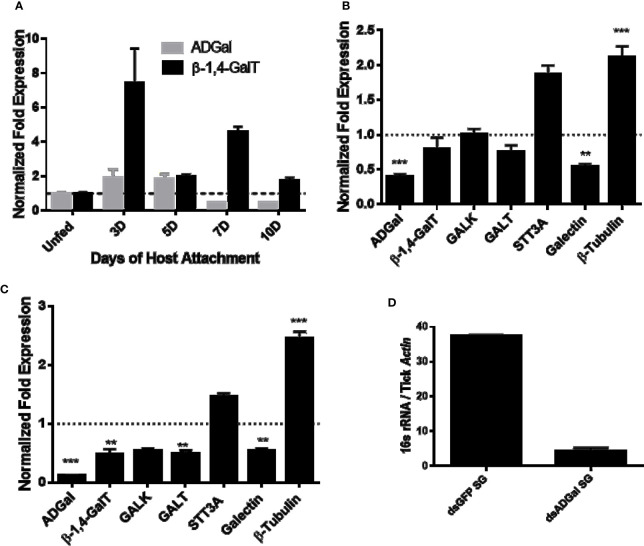
Transcriptional expression in tick tissues. **(A)** Time-dependent transcriptional gene expression of α-D-galactosidase (ADGal) and β-1,4 galactosyltransferase (β- 1,4-GalT) in Amblyomma americanum salivary glands. Fold changes were normalized with the unfed tissue expression. β-actin and histone were used to normalize the gene expression. **(B)** Transcriptional expression of carbohydrate metabolism and transport-related genes in α-D-galactosidase silenced partially blood-fed midguts and, **(C)** salivary glands. β-actin and histone, house-keeping genes were used to normalize the expression against dsGFP treated tissues. β-1,4-GalT, β-1,4 galactosyltransferase; GALK, Galactokinase; GALT, galactosyltransferase; STT3A, Dolichyl-diphosphooligosaccharide-protein glycosyltransferase (**P < 0.01, ***P < 0.001, results are compared by student t-test with unequal variance), **(D)** Total bacterial load partially blood-fed salivary glands injected with dsGFP and dsADGal. Total bacterial load was quantified by qPCR using β-actin as a reference gene. Results are representative of three biological replicates.

Temporal expression in *Am. americanum* salivary glands revealed the α-D-galactosidase expression level increases ~2-fold after tick attachment to the host during the slow feeding phase up to 5 days post-infestation (dpi), and it decreased ~2-fold at 7 dpi and 10 dpi, during the rapid feeding phase (**Figure 1A**). Interestingly, β-1,4-galactosyltransferase transcript level increases ~8-fold after attachment at 3 dpi and remains upregulated (~2 fold at 5 dpi; ~4.5 fold at 7 dpi and ~2 fold at 10 dpi) compared to the unfed salivary glands. Transcriptional expression was normalized against the unfed tick salivary gland. β-actin and histone, two housekeeping genes, were used to normalize the gene expression.

### Gene Silencing and Transcription Expression of Genes Related to Galactose Metabolism

α-D-galactosidase dsRNA injections led to ~65 and ~85% downregulation of ADGal gene expression in both midguts and salivary glands (**Figures 1B, C**), respectively. Transcriptional expression analysis of α-D-galactosidase silenced tick tissues shows the significant downregulation of galectin (~50%), while a significant upregulation of β–tubulin in the midgut (~2-fold increase) and salivary gland tissues (~2.5-fold increase). In the salivary glands, a significant downregulation of β-1,4-GalT of approximately 2-fold and GALT by approximately 2-fold, and a non-significant decrease in galactokinase (GALK) were noted. In addition to that, there was upregulation of STT3A gene in ADGal silenced midgut (~1.6-fold increase) and salivary gland (~0.5-fold increase); however, this change was not statistically significant. Furthermore, there was non-significant downregulation of β-1,4-GT, GALT in ADGal-silenced midgut tissues.

### Impact of α-D-Galactosidase Silencing on Bacterial Load

Total bacterial load quantification assay showed that ~7-fold reduced 16S bacterial load in the salivary gland tissues of *Am. americanum* ticks that received dsADGal injections, compared to dsGFP irrelevant control injected ticks (**Figure 1D**).

### Impact of Gene Silencing on Feeding Success and Tick Engorgement

**Figure 2:**
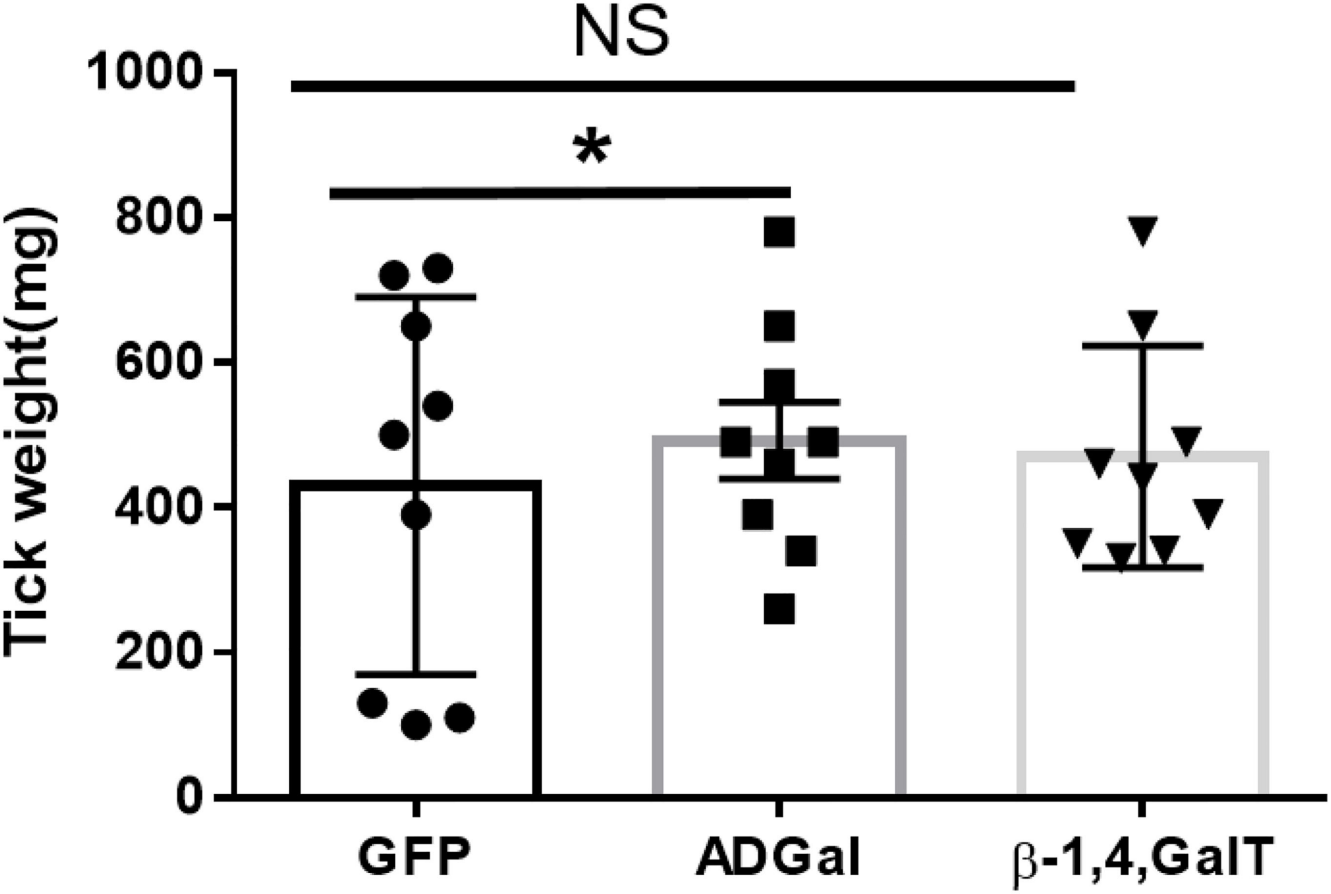
Engorgement weights of ticks treated with dsRNA. Tick weights were taken from mass replete or forcibly removed ticks at four time-points during the bloodmeal after treatment with dsADGal (α-galactosidase), dsβ- 1,4-GT (β-1,4 galactosyltransferase), or dsGFP (irrelevant control) double-stranded RNA (*P < 0.05, results are compared by students t-test with unequal variance, N=9; NS, Non-significant).

To investigate the impact of silencing of α-galactosidase and β-1,4-galactosyltransferase on the tick phenotype, we measured and compared engorged tick weight. Ticks treated with dsADGal engorged faster and weighed more than ticks injected with dsGFP irrelevant control (**Figure 2**). The dsAGS tick weights were significantly (P<0.05) increased at 10 dpi compared with dsGFP control ticks. However, there was no significant difference in tick engorgement in β-1,4-galactosyltransferase-gene-silenced ticks.

### Alpha-Gal Expression in Gene-Silenced Tissues

**Figure 3:**
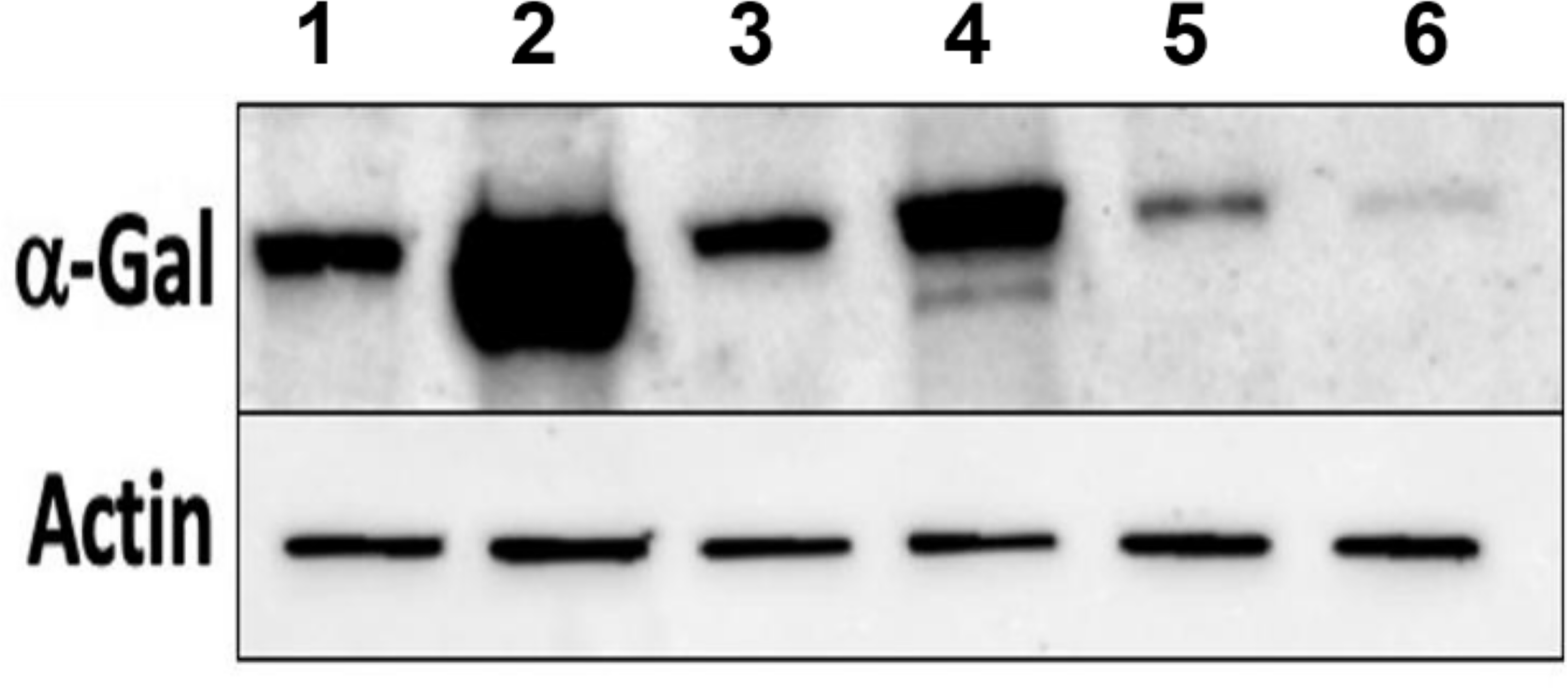
Western blot analysis of α-gal using anti-gal IgM in dsADGal (α-galactosidase) and dsGFP(irrelevant control)-injected partially fed *Am. americanum* salivary glands (pooled SG; N=5 ticks). Lane 1, 5 days post infestation (dpi.) dsADGal salivary glands; Lane 2, 5 dpi dsGFP salivary glands; Lane 3, 7 dpi dsADGal salivary glands; Lane 4, 7 dpi dsGFP salivary glands; Lane 5, 9 dpi dsADGal salivary glands; Lane 6, 9 dpi dsGFP salivary glands.

Salivary glands from 5, 7, and 9 dpi *Am. americanum* ticks injected with dsADGAL and dsGFP irrelevant control RNA were assessed using immunoblotting with an anti-alpha-gal IgM antibody (**Figure 3**). Densitometry analysis was conducted to determine the relative abundance of α-gal in dsADGal-injected tick protein against dsGFP control protein (**Supplementary Figure 3**). Results indicate an ~80% reduction in α-gal in dsADGal salivary gland proteins compared to the dsGFP control. A decrease in α-gal of more than 30% in the 7 dpi dsADGal-injected tick salivary glands, but the 9 dpi salivary glands contained ~10% more α-gal than the dsGFP irrelevant control. While there was no significant difference in α-gal ds β-1,4-GT-injected *Am. americanum* tick salivary gland in comparison to control (**Supplementary Figure 2B**).

### Silencing of α-D-Galactosidase Reduces α-Gal Containing Cross-Reactive Carbohydrate Determinants in Tick Salivary Glands

We performed N-glycan analysis to check the impact of α-D-galactosidase silencing on the abundance of α-gal-containing glycoforms in tick salivary glands. Profile analysis of N-glycan from control samples and dsADGal showed a variety of high mannose, complex type, fucosylated, and alpha-gal containing glycoforms (**Table 1**, **Supplementary Figures 1**, **3**, **4** and **Supplementary Tables 1**–**3**), which were similar in overall trends to the N-glycan profile published previously (Crispell et al., 2019). In addition, the overall α-gal glycoforms’ abundance profile showed that in both samples, the abundance of fucosylated glycoforms was higher than non-fucosylated glycoforms (**Supplementary Tables 1**, **3**, **4**; **Supplementary Figures 3**, **4**). More specifically, overall N-glycans’ abundance containing α-gal glycoforms or moieties in dsGFP-injected control tick salivary gland (pooled salivary glands, N=5 ticks) was 24.02%; however, in the dsADGal-treated salivary glands (pooled salivary glands, N=5 ticks), overall N-glycans containing α-gal moieties were significantly reduced to 2.81%. Among these data, the α-gal having glycoforms at m/z of 2,478 and 2,723 were absent in the dsADGal-treated salivary glands, while abundance of other glycoforms m/z 2,652 and 2,897 was significantly low when compared to control (**Table 1**, **Supplementary Tables 1**, **3**, **4**). These results strengthen the hypothesis that tick α-D-galactosidase is vital in synthesizing or transfer of α-gal to tick salivary glycoproteins.

**Table 1:** N-Glycan analysis on 5dpi *Am. americanum* salivary gland protein extracts.

	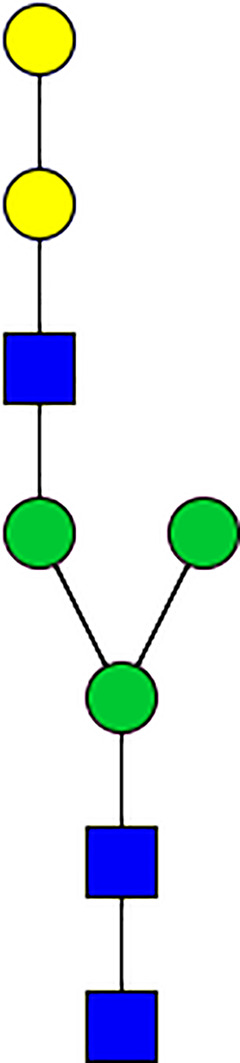	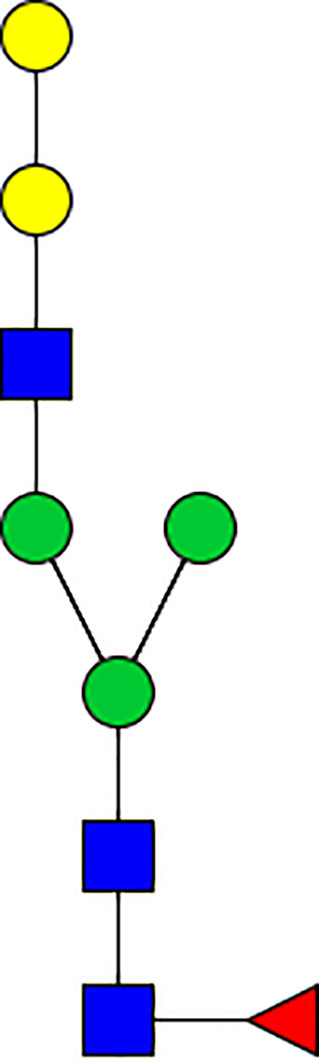	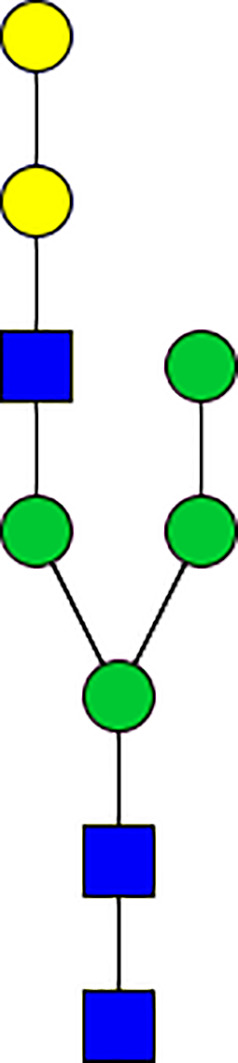	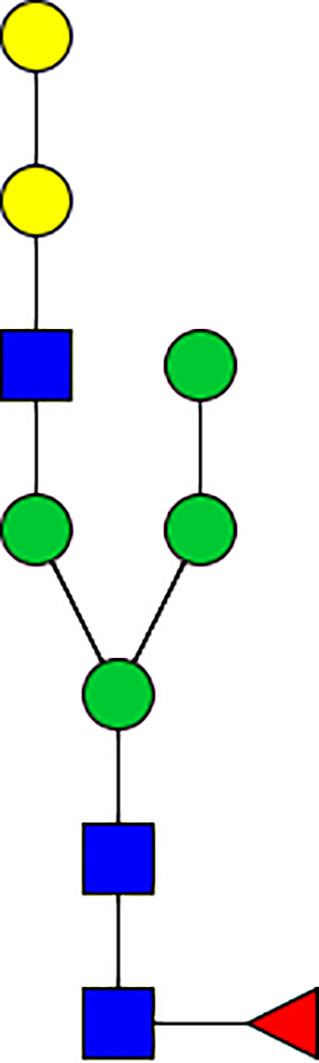	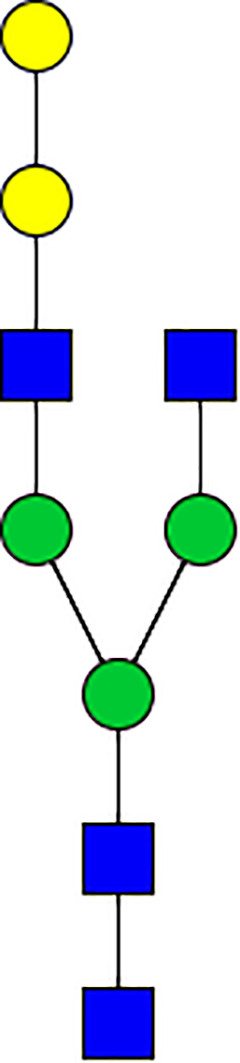	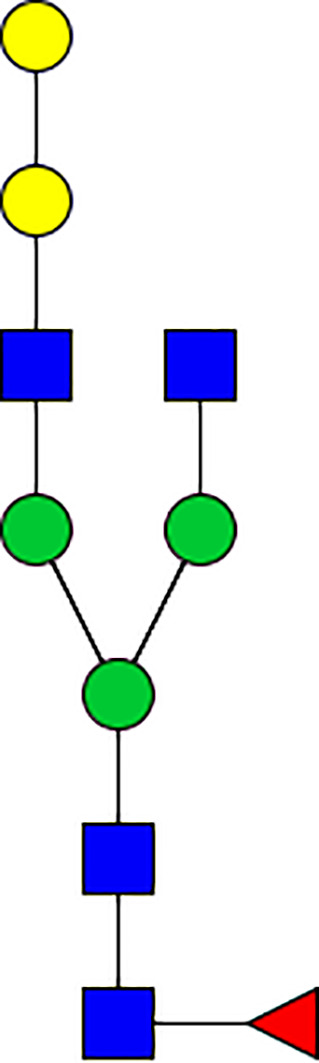	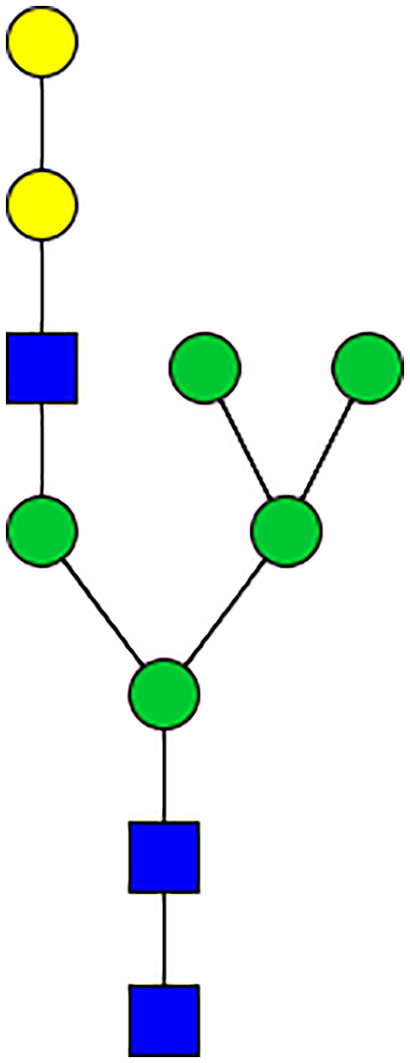	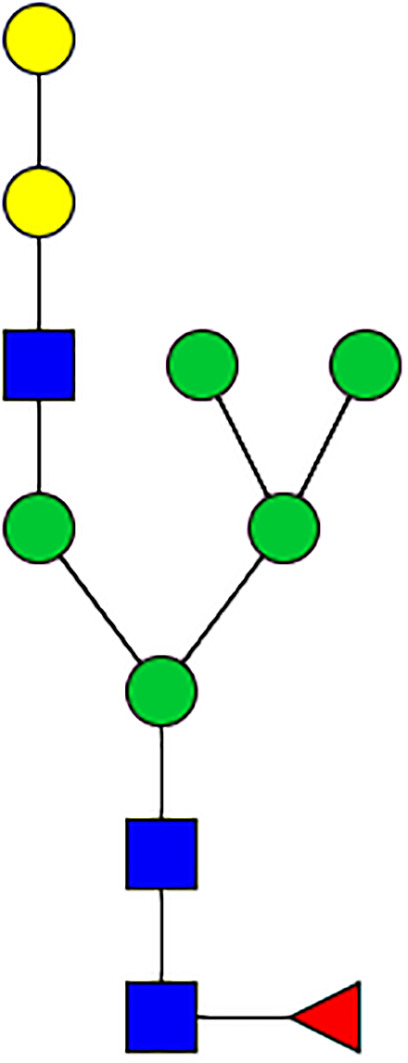	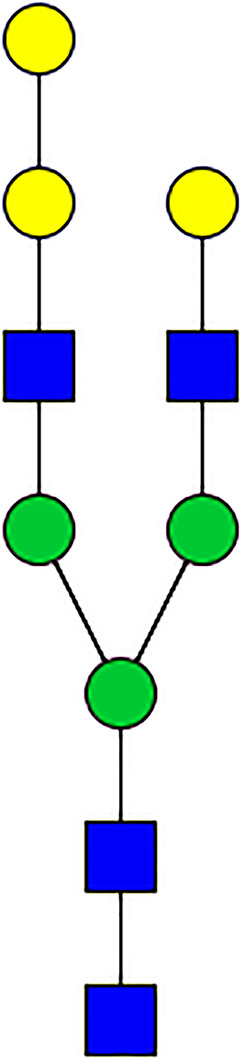	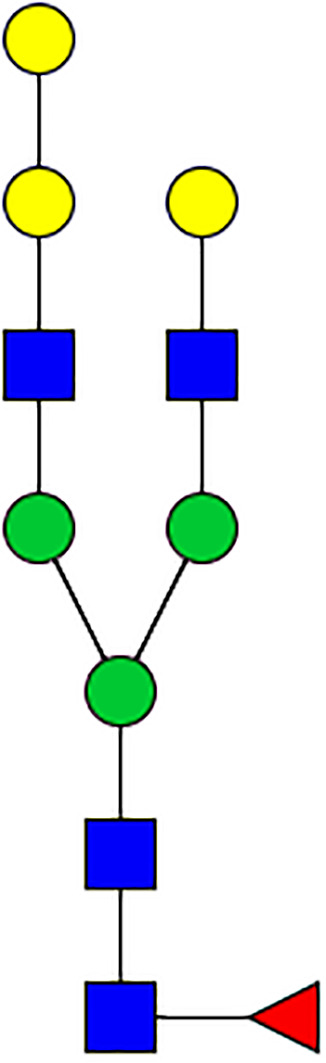
m/z	**1825**	**1999**	**2029**	**2203**	**2070**	**2244**	**2233**	**2407**	**2274**	**2448**
**SG-Control**		0.25%			NO	0.00%				
**SG-KO**	0.11%	0.51%			NO	0.41%				
	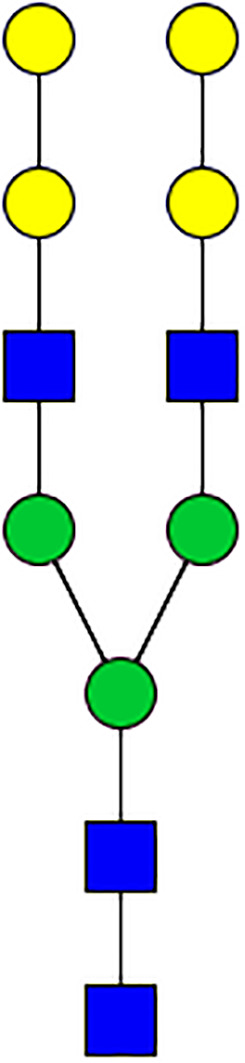	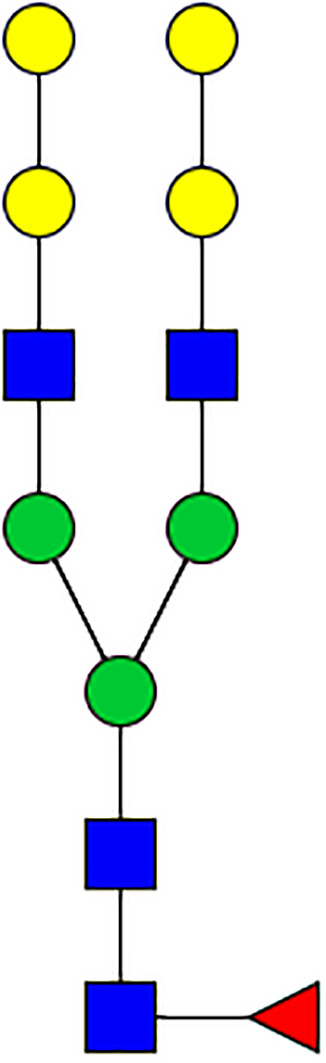	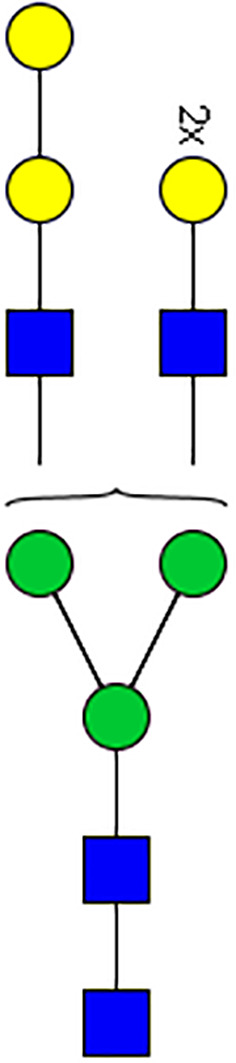	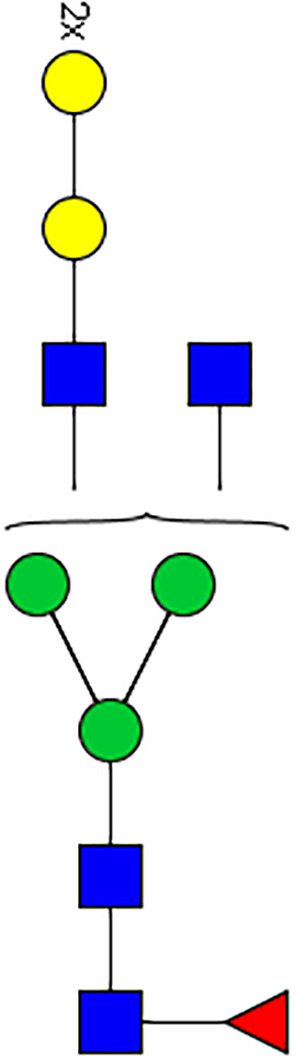	**TOTAL % Alpha-Gal**	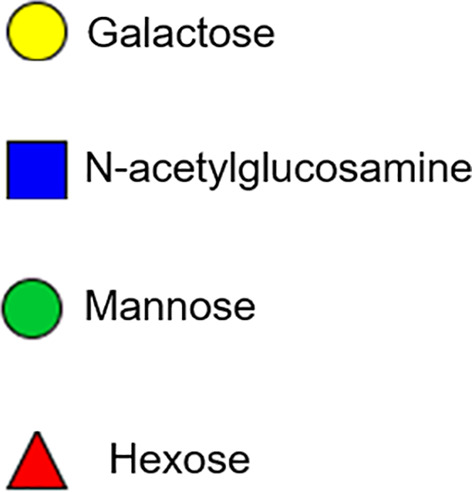				
m/z	**2478**	**2652**	**2723**	**2897**						
**SG-Control**	2.10%	8.94%	4.43%	8.30%	24.02%					
**SG-KO**		1.23%		0.46%	2.81%					

The mass to charge ratio (m/z) signifies different α-gal glycoforms. Total percentage α-gal indicates the percentage sum of different α-gal glycoforms detected in SG-Control dsGFP or irrelevant control) treated and ds α-galactosidase-treated (SG-ADGal KD).Greyed outBoxes indicate that this mass was not detected in that sample. Key: NO: MS/MS fragmentation resulted in 486.23 ion.

### Basophil Activation Test With Tick Salivary Gland Samples

**Figure 4:**
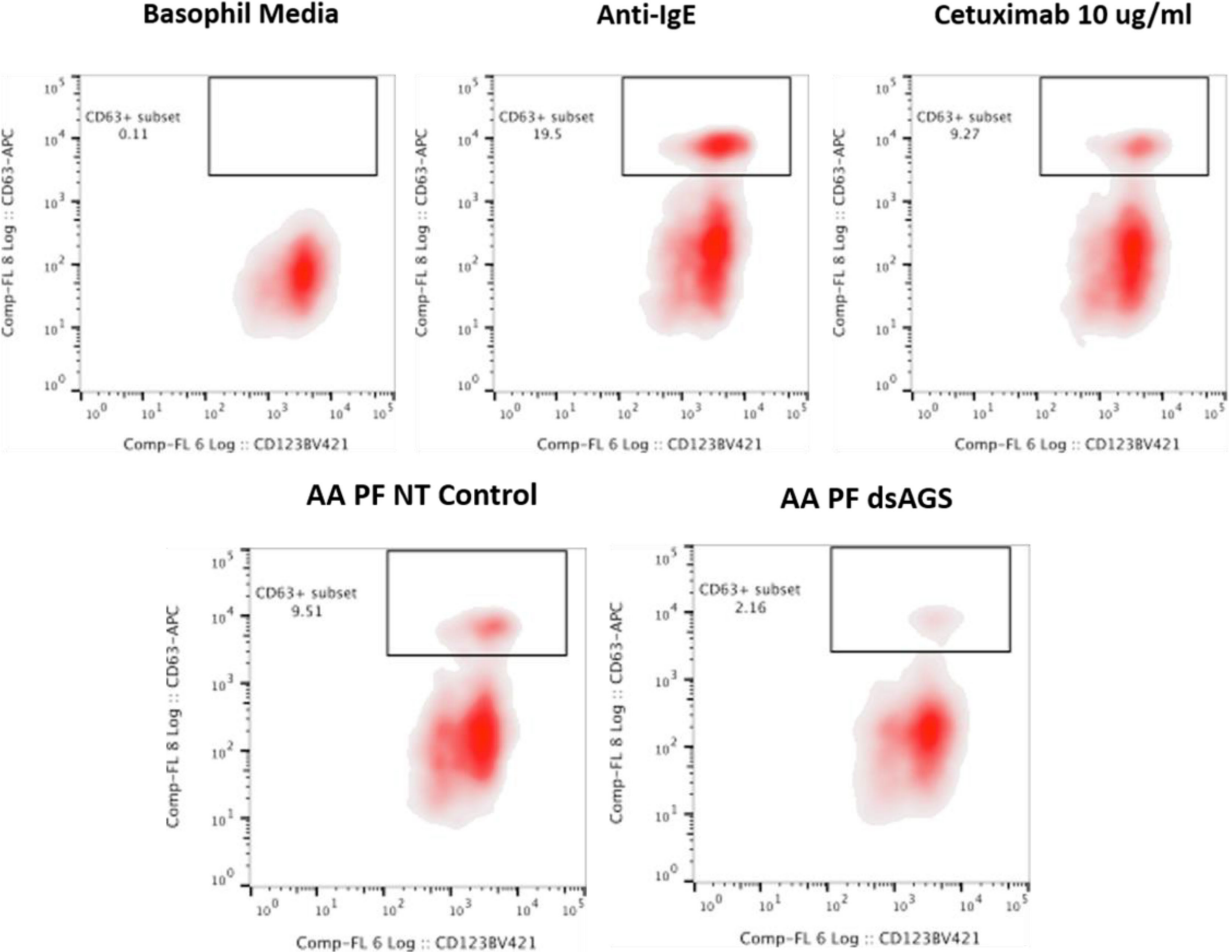
Flow cytometric analysis of human basophil activation by *Amblyomma americanum* salivary proteins (pooled salivary gland, N=5 ticks). Donor basophils from a healthy, non-allergic control subject were stripped of IgE and primed overnight with plasma from a subject with α-gal syndrome (α-gal sIgE = 31.3 IU/ml, total IgE = 233 IU/ml). Sensitized cells were exposed to one of the following stimuli for 30 min: RPMI media, crosslinking anti-IgE antibody (Positive control), α-gal-containing glycoprotein, cetuximab (α-gal positive control), partially-blood-fed *Am. americanum* control, and α-D-galactosidase-gene-silenced salivary gland extracts treated. CD63 expression on lineage-HLA-DR-CD123+CD203c+basophils was assessed by flow cytometry. Results are representative of two biological replicates.

Since the profile of N-glycan demonstrates the presence of α-gal antigen in salivary samples, we sought to analyze the impact of ADGal silencing in basophil activation. In this basophil activation test, the frequency of CD63+-activated donor basophils is lower when PBMCs are stimulated with ADGal-silenced 5 dpi *Am. americanum* salivary gland protein extract in comparison to control 5 dpi *Am. americanum* salivary glands, cetuximab, and Anti-IgE positive control (**Figure 4**). More specifically, we found that the frequency of CD63+ basophils was significantly increased following sensitization with α-gal allergic plasma and stimulation with α-gal-containing tick salivary extract samples from *Am. americanum* (PF SG extract) (p < 0.05 *vs.* media, 4).

Furthermore, ADGal-silenced salivary extract 5 dpi samples (pooled tick SG, N= 5) from *Am. americanum* showed lower mean frequency (mean: 5.6%; SD±4.9) of CD63+ basophil activation. In contrast, the mean frequency of CD63+ activated basophils following stimulation with partially fed 5 dpi salivary extract was 11.4%; SD± 2.6 when the results of all experiments (**Supplementary Table 4**) were compared. Overall, these results suggest a correlation between tick ADGal depletion and potential reduction in the host allergic immune response.

## Discussion

The discovery of α-gal immunoglobulin E (IgE), a central player involved in allergic responses against food containing α-gal antigen such as red meat and in people with a history of tick bite, has caught enormous attention among immunologists and vector biologists (Van Nunen et al, 2009; Crispell et al., 2019; Commins, 2020; Sharma and Karim, 2021). Several research reports have established that antigen-containing α-gal is a key trigger for AGS development (Commins, 2020). Current research focuses on identifying and profiling α-gal antigens in tick saliva and tissues to decipher the interplay between a tick bite and AGS (Cabezas-Cruz et al., 2018; Crispell et al., 2019; Sharma and Karim, 2021). It is still puzzling how the tick acquires and decorates its saliva antigens with α-gal and primes the host immune response. The expression of α-gal antigen in tick saliva is established in different ticks, including *Am. americanum*. However, answer to the questions relating to origin or source of α–gal in a tick and the key mechanistic details of how bite from this tick leads to sensitization of humans against red meat allergy either by triggering the development of memory cell capable of producing α-gal IgE or class switching of IgE because of salivary factor is yet to be clarified. Several pieces of evidence support that the presentation of cross-reactive carbohydrates determinants or the α-gal antigen by ticks during tick feeding is possibly the prime factor for sensitization of humans against α-gal (Crispell et al., 2019; Choudhary et al., 2021). However, how ticks acquire or produce α-gal moiety during feeding remains a complete mystery (Hamsten et al., 2013b; Araujo et al., 2016). There are few working hypotheses regarding the source of α–gal in a tick: (a) α–gal present in a tick is residual or enzymatically modified/cleaved mammalian α–gal containing glycoprotein or glycolipids derived from the mammalian host, (b) α–gal is originating from tick hosted microbes (tick microbiome) capable of expressing α–gal in or possessing the capability to capture cleaved galactose oligosaccharide and glycosylate their own or tick salivary proteins (Sharma and Karim, 2021).

Recently, we have demonstrated (Crispell et al., 2019; Choudhary et al., 2021; Sharma and Karim, 2021) the following: (1) Tick bites, specifically by the lone-star tick, might be solely responsible for stimulating an IgE response to α-gal within the southern and eastern United States. (2) N-linked glycan analysis confirmed the presence of α-gal in the saliva and salivary glands of *Amblyomma americanum* and *Ixodes scapularis*, but *Amblyomma maculatum* contained no detectable quantity. (3) An immuno-proteome approach confirmed the cross-reactivity between tick saliva proteins (allergens) to α-gal antibodies. (4) The presence of antigenic galactose-α-1, 3-galactose (α-gal) epitope activated human basophils as measured by increased expression of CD63. (5) The lone-star tick salivary gland extracts induced AGS in α-Gal^-KO^ mice. An immunoproteome and sialotranscriptome analysis identified several expressed molecules during feeding progression linked with galactose metabolism, N-glycan synthesis, and galactoside transport (Karim and Ribeiro, 2015; Crispell et al., 2019). Surprisingly, the key enzyme, α-1,3-galactosyltransferase (α1,3GalT), synthesized α-gal, remains unidentified in tick genomes and transcriptomes. However, this enzyme is reported to be the key enzyme involved in the synthesis of α1,3-galactose (α-1,3Gal or α-gal) epitopes in several organisms (Galili and Avila, 1999; Galili, 2005; Galili, 2015).

We investigated the role of differentially expressed tick molecules during prolonged blood-feeding on the host, i.e., α-D-galactosidase (glycoside hydrolase/ADGal), an enzyme that catalyzes the breakdown of galactose from glycoproteins or glycolipids, and β-1,4-galactosyltransferase (β-1,4-GalT), an enzyme that is involved in the synthesis of Galβ1-4-GlcNac-disaccharide unit of glycoconjugates (Hennet, 2002; Calhoun et al., 1985). We hypothesized that the expression of these genes during prolonged feeding can impact overall α–gal expression, expression of key genes of galactose catabolism (Leloir pathway), N-glycan synthesis, galectin (a molecule involved in the transport of galactose containing oligosaccharides) (Huang et al., 2007; Díaz-Alvarez and Ortega, 2017), and β-tubulin (a glycosylated marker molecule). Leloir pathway (**Supplementary Figure 1**) is the predominant route of galactose metabolism, and products of this pathway generate key energy molecules as substrate, i.e., UDP-galactose vital for N-glycan synthesis (Karim and Ribeiro, 2015; Freeze et al., 2017). More specifically, selected key molecules of the Leloir pathway included in this study were galactokinase (GALK), galactose-1-phosphate uridyltransferase (GALT). GALK modifies galactose to create a molecule called galactose-1-phosphate, which can be further added to build galactose-containing proteins or fats (McAuley et al., 2016). The GALT catalyzes the second step of the Leloir pathway (**Supplementary Figure 1**), converting galactose into glucose (Wong and Frey, 1974). In addition to that, another molecule, AamSigP-24522 putative dolichyl-diphosphooligosaccharide protein glycosyltransferase (STT3A), which is reported to be involved in the early stage of cotranslational N-glycosylation of target protein (Cherepanova and Gilmore, 2016), was also included in the study to check the impact of silencing of ADGal and β-1,4-GalT in the initiation of N- linked glycosylation process.

Furthermore, in this study, the impact of ADGal and β-1,4-GalT silencing on glycosylated molecule and molecule involved in transport, and the expression of β-tubulin and galectin were tested. In addition to that, in this study, we analyzed the expression of α-gal antigen in salivary glands of *Am. americanum* across the different feeding stages, and we observed that α-gal antigens in partially blood-fed are significantly upregulated (3–5 days) and gradually downregulated as feeding transitions towards the fast-feeding stage and repletion. Such a concurring trend of differential expression of ADGal and α-gal antigen expression in a tick salivary gland across the feeding stages points towards the role of sialome-switch in tick’s α-gal signature, and its potential significance during hematophagy is intriguing (Karim and Ribeiro, 2015). One plausible explanation of such differential expression of α-gal antigen, especially during partially feeding stages, might be one vital strategy adapted by ticks to evade host response by mimicking a vertebrate glycan. A similar molecular mimicry mechanism is also reported in nematodes and protozoans (Duffy et al., 2006). Studies have suggested that α-gal deficient host develops anti-α-gal IgE antibodies when bitten by α-gal producing tick species and recruitment of basophils and mast cells occurs to the bite site, as a potential defense by host against tick saliva antigens (Chandrasekhar et al., 2020; Sharma and Karim, 2021). While α-gal expression in tick salivary proteins or cement components offer an advantage to the tick during slow feeding phase, it diminishes during fast feeding phase possibly due to the naturally circulating anti-gal response from the host. Even though ticks’ ability to express α-gal is evident, the central question regarding tick’s inherent ability to express α-gal remains enigmatic because of the lack of α-1,3 galactosyltransferase sequence in tick databases. To date, there are 1,776 genomes from vertebrates available at the NCBI genome site (https://www.ncbi.nlm.nih.gov/genome/?term=vertebrata) and 2,513 arthropod genomes, 11 of which are from ticks. Likewise, the silencing of the ADGal gene in both midgut and salivary gland tissues showed an interesting compensatory expression pattern of genes involved in galactose metabolism (**Supplementary Figure 1**). The transcript levels of β-1, 4-galactosyltransferase, galactose-1-phosphate uridyltransferase (GALT), and galactose binding transport protein galectin were significantly downregulated, while the transcript level of β-tubulin was significantly upregulated. GALK, GALT, and galectin downregulation may be negative feedback responses due to the reduction of galactose-containing oligosaccharides caused by silencing of ADGal. While putative dolichyl-diphosphooligosaccharide protein glycosyltransferase subunit STT3A gene was upregulated, upregulation was not statistically significant, probably due to the compensatory effect from the redundant molecule. Since the temporal expression pattern of β-1,4-GalT coincided with α-gal antigen expression, we also carried out a functional study using RNAi.

Regardless of significant silencing of β-1,4-GT gene in salivary gland tissues, the transcript level of ADGal, GALK, GALT, STT3A, galectin, β-tubulin, as well as α-gal-expression in *Am. americanum* showed no significant change. These results suggest β-1, 4-GalT does not contribute to the α-gal signature in *Am. americanum*. Intriguingly, three variants of galactosyltransferase genes, i.e., β4galt-7, α4galt-1 and α4galt-2 from β-1,4-GalT, and α-1,4-GalT family are shown to be involved in the α-gal synthesis pathway (Cabezas-Cruz et al., 2018). However, search for *Ix. scapularis* homologs in *Am. americanum* failed to yield any CDS in the existing NCBI transcriptomic database. Since the genome sequence of *Am. americanum* is not available so far, hence, it will be challenging to conclude the absence of such important genes. However, a clear difference in the pattern of α-gal expression and N-glycan signatures among ticks *Ixodes scapularis*, *Am. americanum*, and *Am. maculatum* also indicates a possible evolutionary difference of glycosylation or glycan modification machinery and the potential role of tick’s extrinsic and intrinsic factors linked to glycosylation or glycan profile (Crispell et al., 2019, Sharma and Karim, 2021).

Since ADGal is an enzyme responsible for releasing α-gal from substrates, it is differentially expressed across the feeding stages, indicating tick may be utilizing it for removing α-gal from host-derived glycosylated lipid or proteins. To answer the question that α-D-galactosidase plays a critical role in galactose metabolism and α-gal signature in the tick tissues, we silenced the ADGal gene and performed N-Glycan analysis. The results indicated a significant reduction in the overall abundance of N-glycans containing α-gal glycoforms in tick samples. Basophil activation test using control and ADGal Salivary Gland Extract (SGE) showed a reduction in the frequency of activated basophil compared to control. These results further confirmed the N-Glycan analysis in the gene-silenced salivary glands. The engorgement weights of dsADGal ticks compared to control ticks showed a significant difference, and gene-silenced ticks gained weight faster than control ticks. This phenotype could result from the tick’s compensatory mechanisms to losing a key galactose metabolizing molecule. The transcriptional expression data suggest the silencing of ADGal inhibits the tick’s Leloir galactose metabolism pathway by downregulating the expression of intermediate enzymes, GALK and GALT, and correlates with differential expression of other galactose-modifying genes including, β1-4, galactosyltransferase, galectin. The Leloir pathway ultimately channels into glycolysis and produces ATP. The ATP was not quantified in these experiments. Still, the downregulation of Leloir pathway genes and decrease in α-gal may have led to the tick producing less energy and compensating it by imbibing high quantity of blood meal (Raven and Johnson, 1995). Our findings support the functional role of ADGal in the tick’s energy utilization.

A 6-fold decreased total bacterial load in ADGal silenced partially-blood fed tick tissues supports the hypothesis that reduction in ADGal enzyme activity leads to reduction in availability of free galactose or glucose and reduces the total bacterial load within the ticks. These results also support the functional role of ADGal in maintaining microbial homeostasis within the tick salivary glands. These findings further warrant investigations to examine the role of bacterial communities in AGS because of their possible role in manipulating ticks’ metabolic activity and glycosylation machinery. Microbiome homeostasis within the tick is critical in the context of the α-gal syndrome (AGS). Galactose is vital for microbes not only as an energy molecule but also as a key molecule required to produce glycosylated exopolysaccharides or lipopolysaccharides (LPS), a potential α-gal antigen (Chai et al., 2012). In addition, the presence of specific microbes in the tick vector can also affect metabolome, especially galactose. One recent study of tick-*Borrelia* interaction found that relative abundance of galactose was significantly reduced in *Borrelia burgdorferi* and *Borrelia mayonni*–infected ticks (Hoxmeier et al., 2017). Likewise, an earlier report on the role of galactose and Leloir pathway genes, especially galactosyltransferase, established that galactose and bacterial galactosyl transferases are vital in biofilm formation for colonization of bacteria (Chai et al., 2012). Hamadeh et al. (1995) demonstrated glycosylation of human erythrocytes (RBCs) *in vivo* by a bacterial α1,3 galactosyl transferase enzyme. Another recent study demonstrated that the tick-borne pathogen *Anaplasma phagocytophilum* increases α-gal antigen in IRE *Ix. ricinus* tick cells (Cabezas-Cruz et al., 2018). Montassier et al. (2019) reported the presence of α1,3-galactosyltransferase bacterial sequences in the human gut microbiome shotgun sequencing project (Montassier et al., 2019). The microbes from Rizobiaceae and Caulobacteriaceae families, which are also reported in lone star tick microbiome were found to possess a novel lipid A a-(1-1)-GalA transferase gene (rgtF) (Brown et al., 2013; Kumar et al., 2021). These findings provided a supporting basis for the hypothesis that a glycosylated lipid could be one augmenting factor for sensitization. Bacteria utilize this machinery to synthesize exogenous lipopolysaccharides (LPS), hence lipid A a-(1,1)-GalA transferase, which could be necessary for an α-gal antigen development (Brown et al., 2013; Del Moral and Martínez-Naves, 2017). Considering all these facts, it is inferred that the microbiome could also be one factor involved in the sensitization against α-gal while the tick is feeding on the host.

## Conclusion

The results from this study led to the conclusion that the (a) tick α-D-galactosidase and β-1,4-galactosyltransferase are important enzymes that are differentially expressed in salivary glands, and tick possibly utilizes these enzymes to cleave α-gal from host proteins or lipids and recycle or add in its proteins during hematophagy; (b) α-D-galactosidase silencing reduces N-glycan signature (α-gal moieties) in the tick salivary glands; (c) α-gal expression is high in partially-blood-fed ticks and gradually decreases towards the end of feeding progression; (d) β-1,4-galactosyltransferase downregulates galactose catabolism, however silencing does not affect overall α-gal expression or signature in salivary glands; (e) α-D-galactosidase silencing also significantly reduces the microbial load in the salivary glands, indicating its role in microbial homeostasis. Overall, α-D-galactosidase is an important enzyme, and its expression is directly linked to endogenous production or expression of α-gal in the ticks during hematophagy. The results presented here add new insights into understanding the role of vital tick intrinsic factors involved in synthesizing or recycling of α-gal and sensitizing host against α-gal during hematophagy.

## Data Availability Statement

The datasets presented in this study can be found in online repositories. The names of the repository/repositories and accession number(s) can be found in the article/**Supplementary Material**.

## Ethics Statement

The animal study was reviewed and approved by the Institutional Animal Care and Use Committee (IACUC) of the University of Southern Mississippi, Hattiesburg, MS 39406, USA.

## Author Contributions

Conceptualization: SK. Methodology: SS, GC, AM, CC, JL, SCh, SCo, SK. Data curation: SS, GC, SCh, SCo, SK. Funding acquisition: SCo, SK. Investigation: SS, GC, SCh, SCo, SK. Project administration: SK. Resources: SCo, SK. Supervision: SK. Validation: SS, SCo, SK. Writing, original draft: SS, GC, SK. Writing, review and editing: SS, SCo, SK. All authors contributed to the article and approved the submitted version.

## Funding

This research was principally supported by USDA NIFA award #2017-67017-26171, the National Institutes of Allergy and Infectious Diseases award, RO1 AI35049; the Mississippi INBRE [an institutional Award (IDeA) from the National Institute of General Medical Sciences of the National Institutes of Health under award P20GM103476]. The funders played no role in the study design, data collection and analysis, decision to publish, or preparation of the manuscript.

## Conflict of Interest

The authors declare that the research was conducted in the absence of any commercial or financial relationships that could be construed as a potential conflict of interest.

## Publisher’s Note

All claims expressed in this article are solely those of the authors and do not necessarily represent those of their affiliated organizations, or those of the publisher, the editors and the reviewers. Any product that may be evaluated in this article, or claim that may be made by its manufacturer, is not guaranteed or endorsed by the publisher.

## Supplementary Material

Supplementary Figure 1 Proposed galactose metabolism pathway of *Amblyomma americanum.*

Supplementary Figure 2 Quantification of relative abundance of α-gal in dsADGal (α-galactosidase) and dsGFP (irrelevant control)-injected partially-fed *Am. americanum* salivary glands.

Supplementary Figure 3 FTMS of N-glycans observed in control salivary gland

Supplementary Figure 4 FTMS of N-glycans observed in KO salivary gland

Supplementary Figure 5
**(A)** Transcriptional expression of major galactose metabolism, N-glycan synthesis- and transport-related genes against ß-1,4 galactosyl transferase silencing in 5-day partially feed salivary gland (SG) of *Am. americanum*. Actin and histone were used as housekeeping genes, and expression was normalized against irrelevant control (GFP dsRNA). Abbreviation: ADgal, α-D-galactosidase; ß-1,4 GT, ß-1,4 galactosyl transferase; GALK, Galacto kinase; GALT, galactosyl transferase; STT3A, Dolichyl-diphosphooligosaccharide-protein glycosyltransferase (***P<0.001, student t test). **(B)** Detection of α-gal in ds ß-1,4GT, dsGFP, and non-treated injected partially-fed tick salivary glands. Results are representive of five biological replicates.

Supplementary Table 1 Summary of potential α-Gal Glycoforms Determined by MS/MS Analysis.

Supplementary Table 2 FTMS of N-Glycans observed in control salivary glands.

Supplementary Table 3 FTMS of N-glycans observed in α-D-galactosidase-silenced salivary glands.

Supplementary Table 4 Frequency of basophil activation. Miscellaneous Supplementary Figures FTMS of all N-glycans observed in KO salivary and control gland.
