# Supplementary material for: Alpha-Gal Syndrome: Involvement of Amblyomma americanum α-D-Galactosidase and β-1,4 Galactosyltransferase Enzymes in α-Gal Metabolism
Source: Front Cell Infect Microbiol. 2021 Dec 1;11:775371. doi: 10.3389/fcimb.2021.775371 (PMC8671611; doi:10.3389/fcimb.2021.775371)
Supplement: Supplementary Figure 1 — Proposed galactose metabolism pathway of Amblyomma americanum. [file Image_1.pdf]

Supplementary information

Supplementary figure1 – Proposed galactose metabolism pathway of *Amblyomma americanum.*

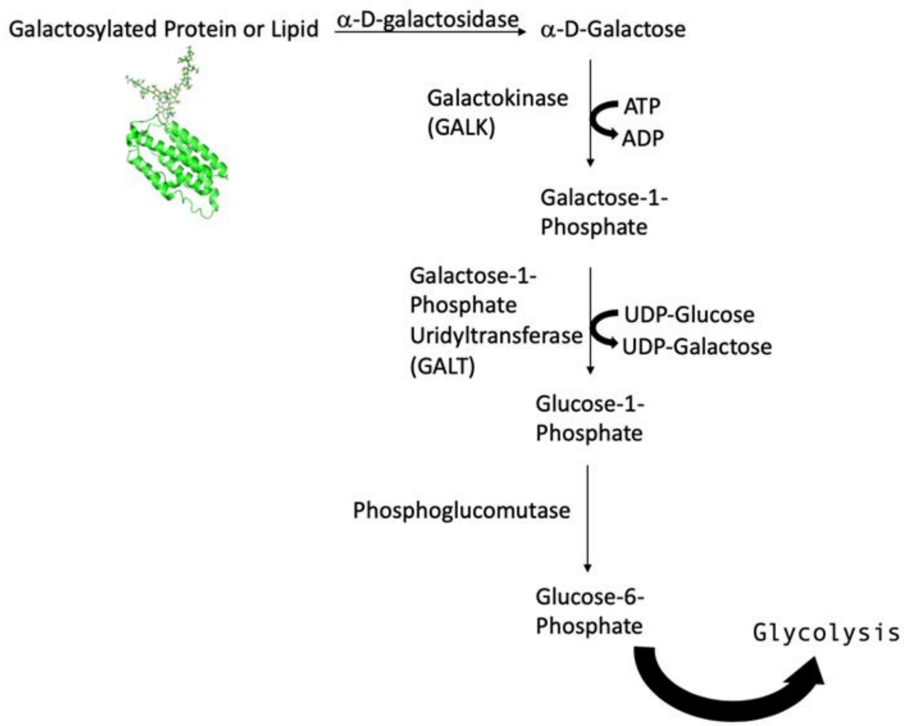

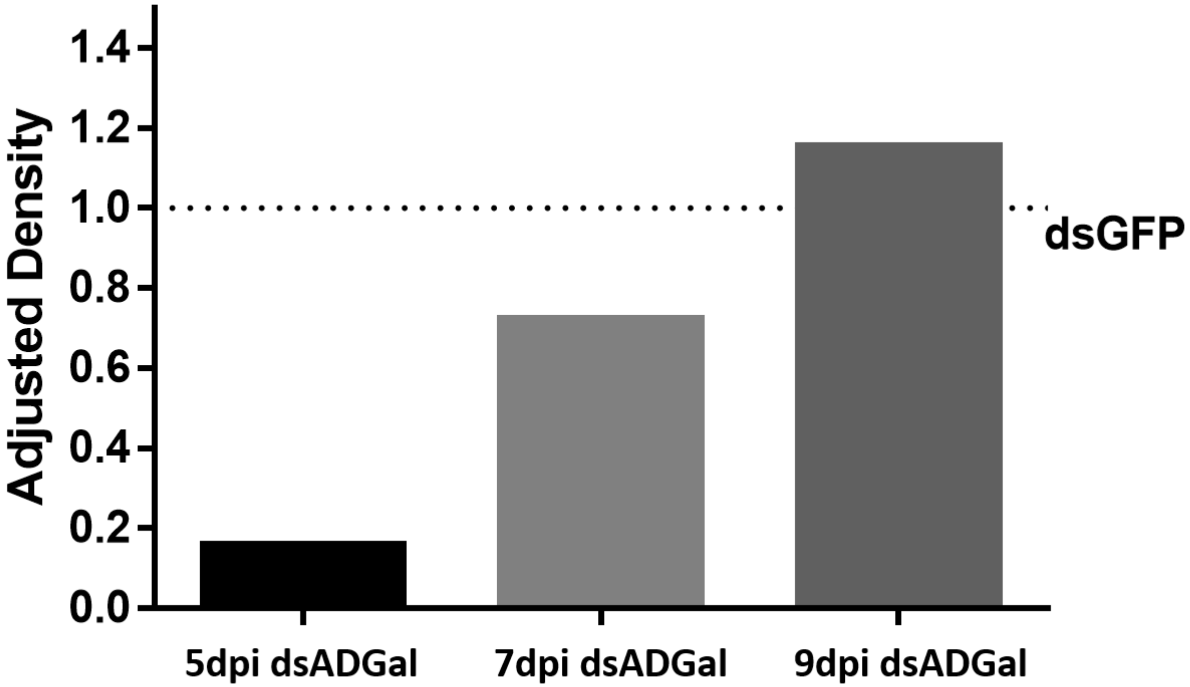

Supplementary figure 2: Quantification of relative abundance of α-gal in dsADGal (α-galactosidase) and dsGFP(irrelevant control) injected partially-fed *Am. americanum* salivary glands.

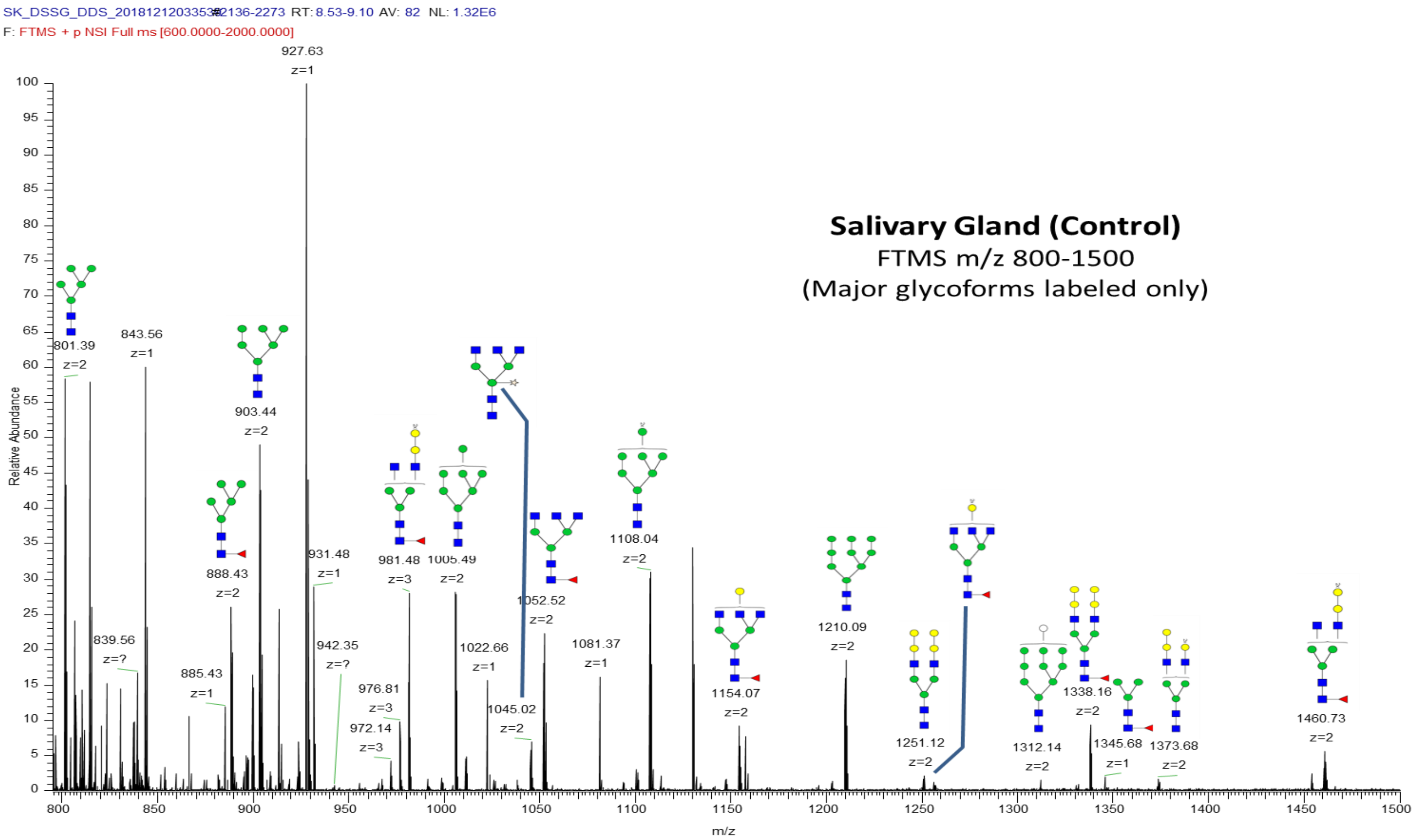

Supplementary figure 3: FTMS of N-glycans observed in control Salivary Gland

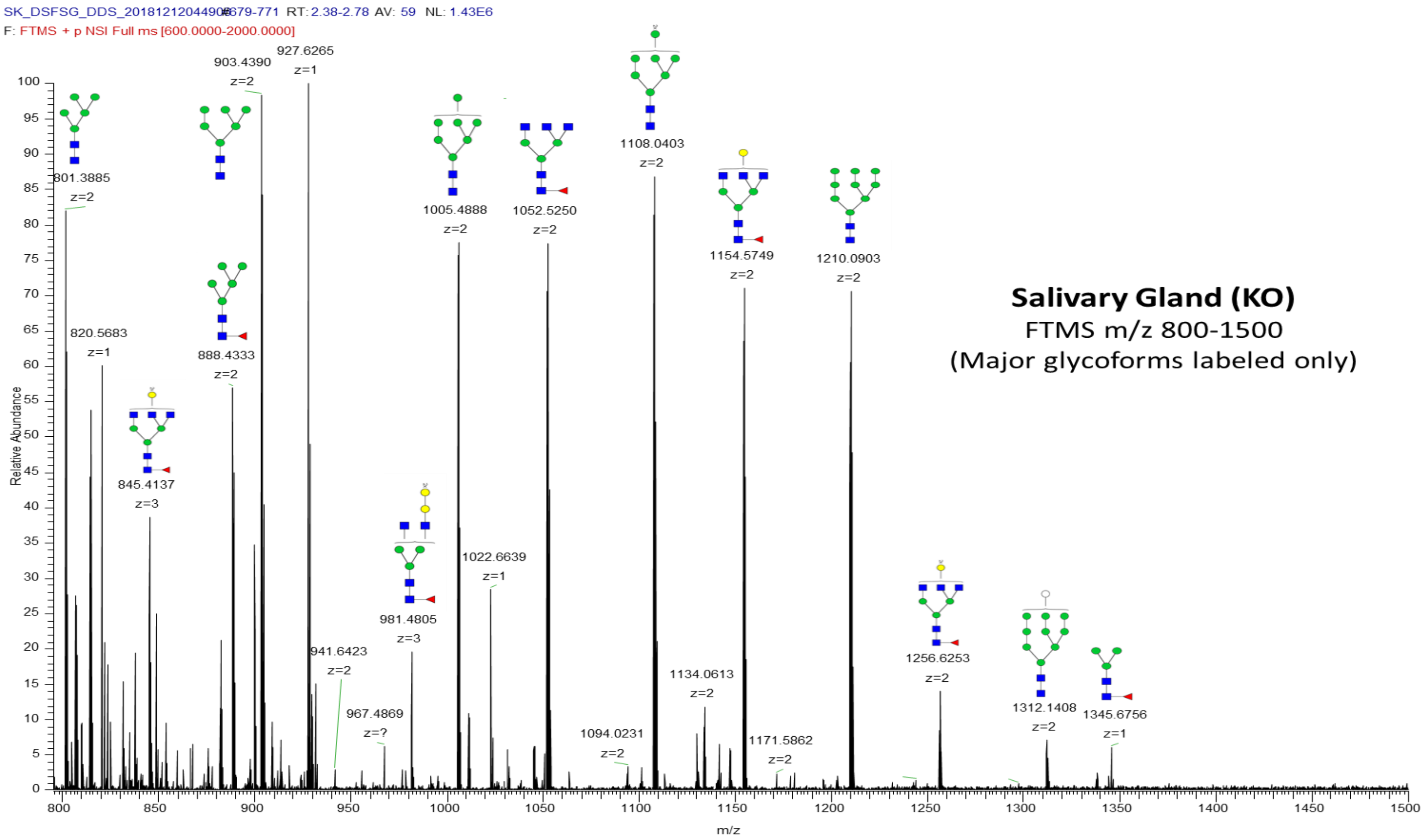

Supplementary figure 4: FTMS of N-glycans observed in KO Salivary Gland

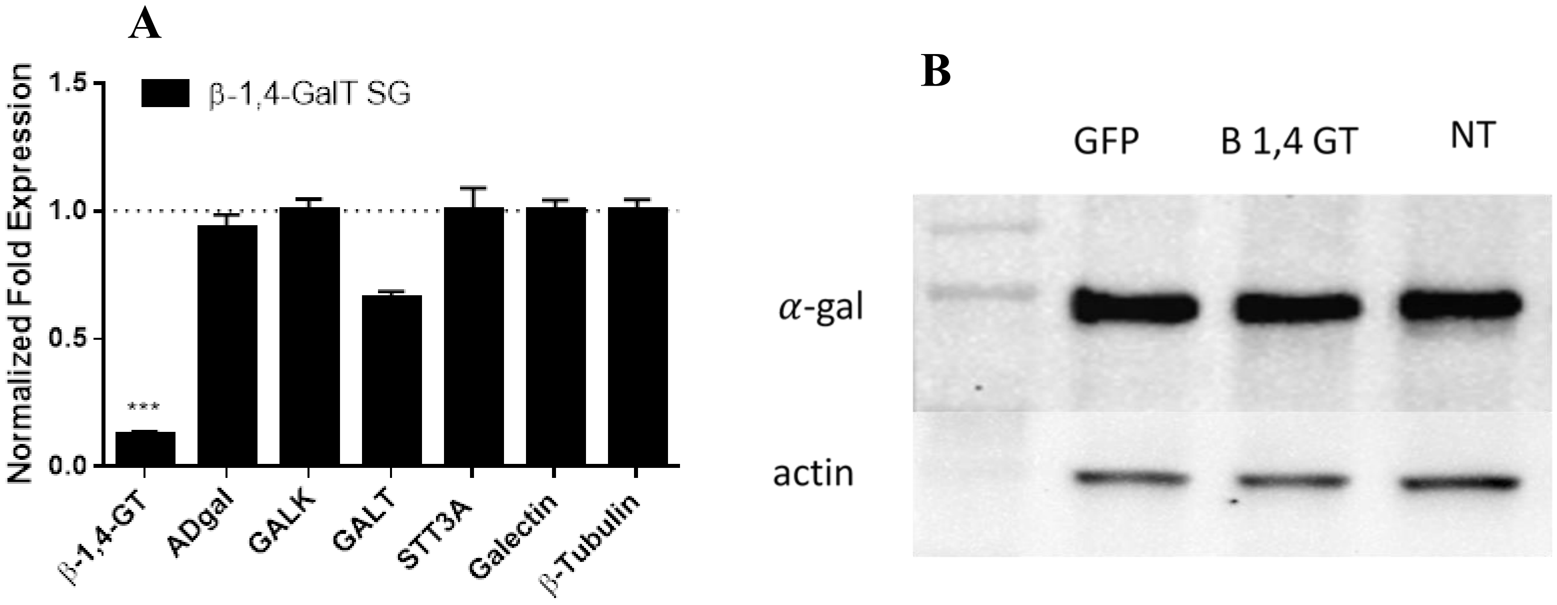

Supplementary figure 5: (A) Transcriptional expression of major galactose metabolism, N-glycan synthesis and transport related genes against ß- 1,4 galactosyl transferase silencing in five day partially feed salivary gland(SG) of *Am. americanum*. Actin and Histone was used as house keeping gene and expression was normalized against irrelevant control (GFP dsRNA). Abbreviation: ADgal α-D-galactosidase ,ß- 1,4 GT: ß- 1,4 galactosyl transferase; GALK: Galacto kinase; GALT galactosyl transferase; STT3A: Dolichyl-diphosphooligosaccharide--protein glycosyltransferase(***P<0.001, student t test) (B) Detection of α -gal in ds ß-1,4GT, dsGFP and non treated injected (NT) partially-fed tick salivary glands. Results are representive of five biological replicates.

Miscellaneous supplementary figures/Data: FTMS of all N-glycans observed in KO Salivary and control Gland

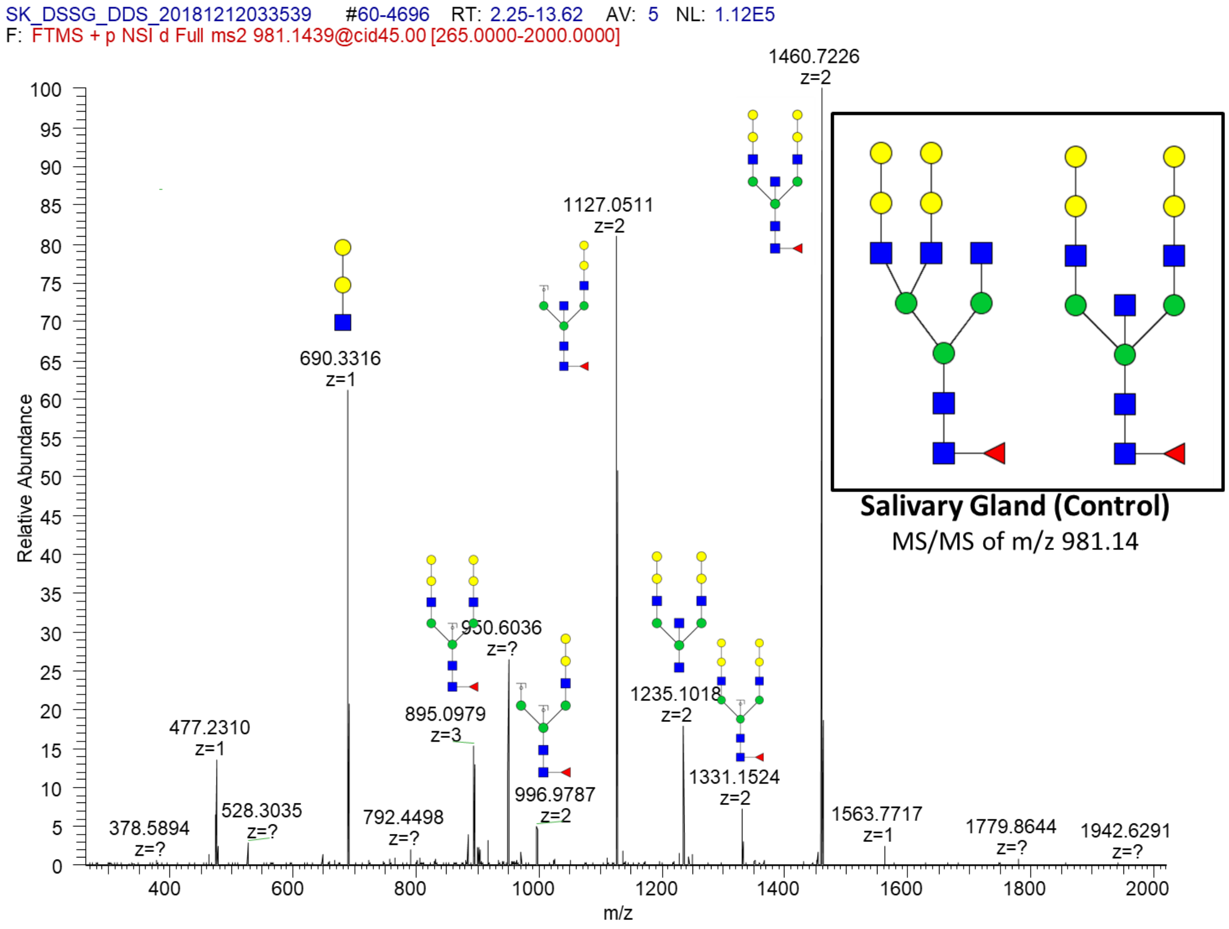

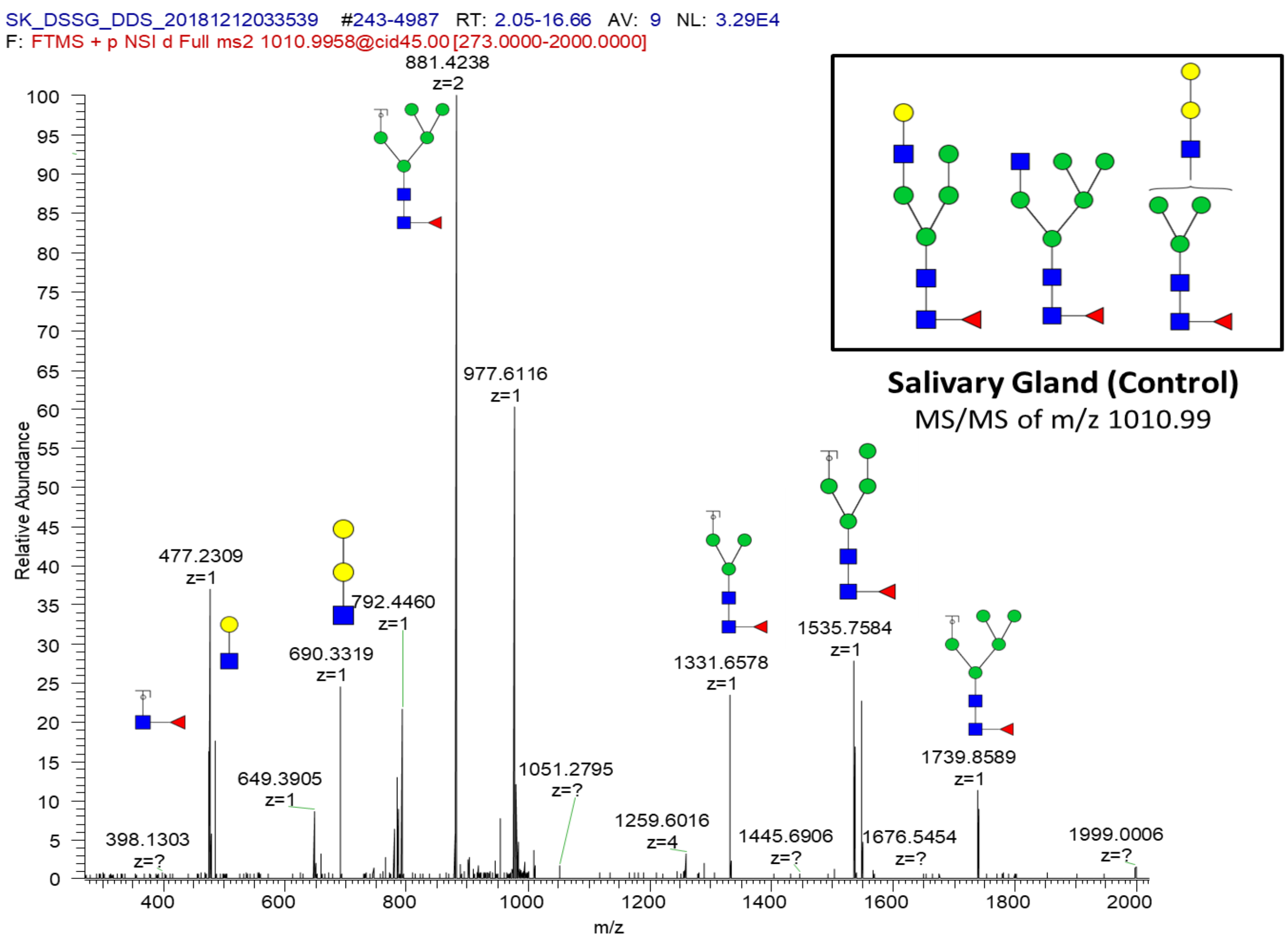

SK_DSSG_DDS_20181212033539 #243-4987 RT: 2.05-16.66 AV: 9 NL: 3.29E4
F: FTMS + p NSI d Full ms2 1010.9958@cid45.00 [273.0000-2000.0000]
881.4238 z=2
977.6116 z=1
477.2309 z=1
792.4460 z=1
690.3319 z=1
649.3905 z=1
398.1303 z=?
1051.2795 z=?
1259.6016 z=4
1331.6578 z=1
1445.6906 z=?
1535.7584 z=1
1676.5454 z=?
1739.8589 z=1
1999.0006 z=?
Relative Abundance
m/z
Salivary Gland (Control)
MS/MS of m/z 1010.99

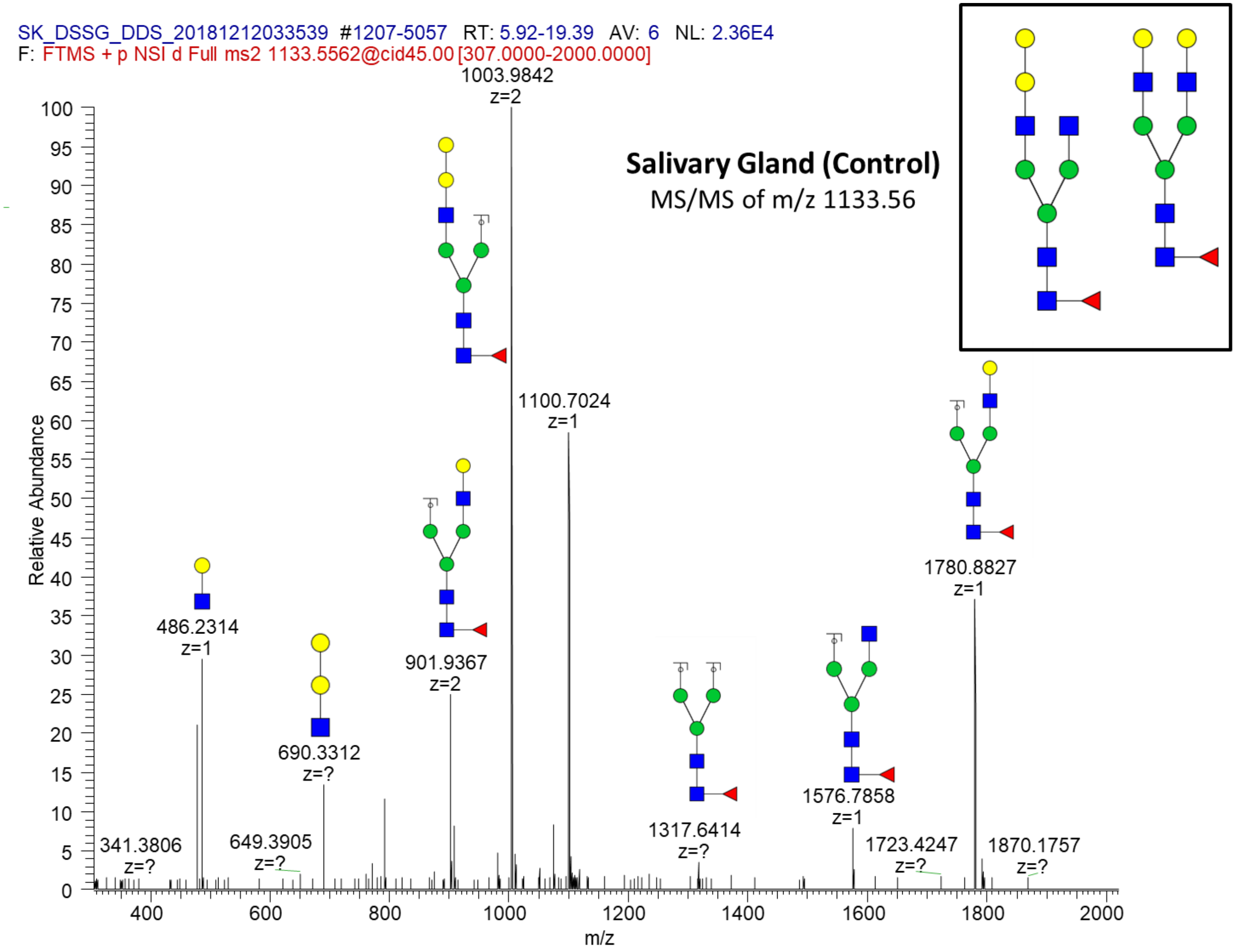

SK_DSSG_DDS_20181212033539 #1207-5057 RT: 5.92-19.39 AV: 6 NL: 2.36E4
F: FTMS + p NSI d Full ms2 1133.5562@cid45.00 [307.0000-2000.0000]
Salivary Gland (Control)
MS/MS of m/z 1133.56
Relative Abundance
m/z
1003.9842 z=2
1100.7024 z=1
1780.8827 z=1
486.2314 z=1
690.3312 z=?
901.9367 z=2
1317.6414 z=?
1576.7858 z=1
341.3806 z=?
649.3905 z=?
1723.4247 z=?
1870.1757 z=?

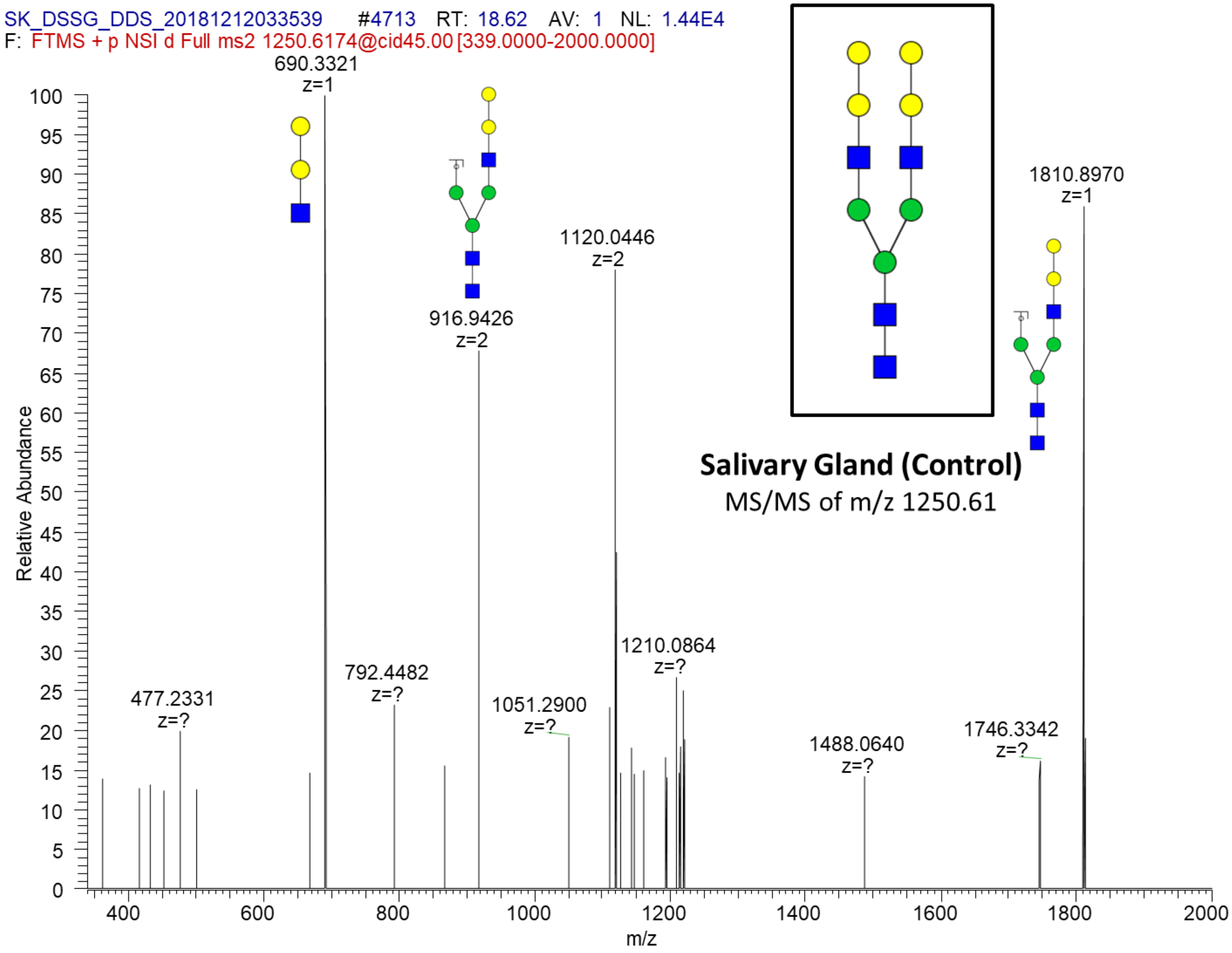
SK_DSSG_DDS_20181212033539 #4713 RT: 18.62 AV: 1 NL: 1.44E4
F: FTMS + p NSI d Full ms2 1250.6174@cid45.00 [339.0000-2000.0000]
690.3321 z=1
916.9426 z=2
1120.0446 z=2
1810.8970 z=1
477.2331 z=?
792.4482 z=?
1051.2900 z=?
1210.0864 z=?
1488.0640 z=?
1746.3342 z=?
Relative Abundance
m/z
Salivary Gland (Control)
MS/MS of m/z 1250.61

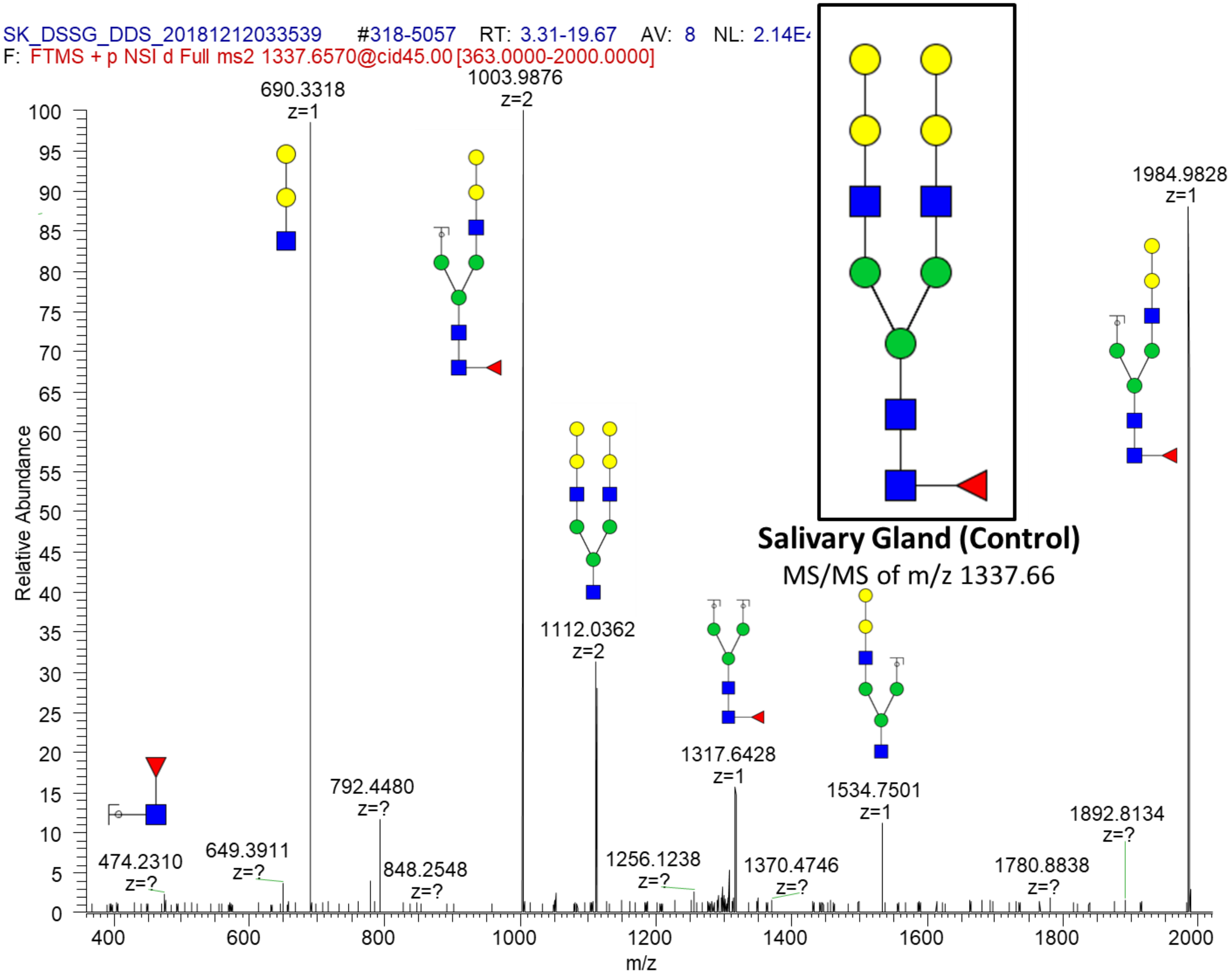

SK_DSSG_DDS_20181212033539 #318-5057 RT: 3.31-19.67 AV: 8 NL: 2.14E4
F: FTMS + p NSI d Full ms2 1337.6570@cid45.00 [363.0000-2000.0000]
Relative Abundance
m/z
690.3318 z=1
1003.9876 z=2
1984.9828 z=1
1112.0362 z=2
1317.6428 z=1
1534.7501 z=1
792.4480 z=?
474.2310 z=?
649.3911 z=?
848.2548 z=?
1256.1238 z=?
1370.4746 z=?
1780.8838 z=?
1892.8134 z=?
Salivary Gland (Control)
MS/MS of m/z 1337.66

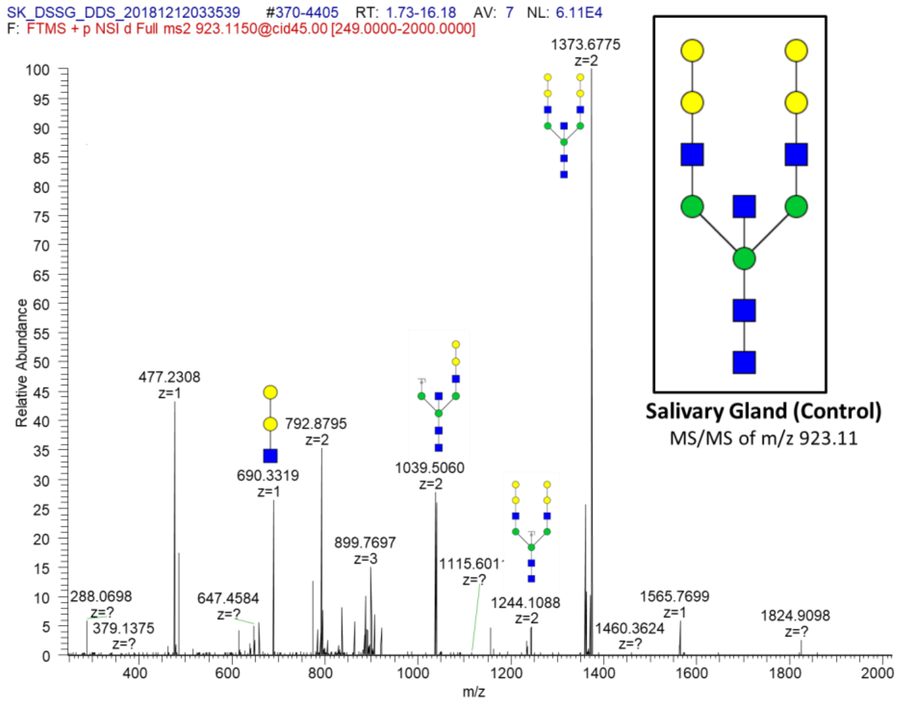
SK_DSSG_DDS_20181212033539 #370-4405 RT: 1.73-16.18 AV: 7 NL: 6.11E4
F: FTMS + p NSI d Full ms2 923.1150@cid45.00 [249.0000-2000.0000]
Relative Abundance
m/z
1373.6775 z=2
477.2308 z=1
792.8795 z=2
690.3319 z=1
1039.5060 z=2
899.7697 z=3
1115.601 z=?
288.0698 z=?
647.4584 z=?
379.1375 z=?
1244.1088 z=2
1565.7699 z=1
1460.3624 z=?
1824.9098 z=?
Salivary Gland (Control)
MS/MS of m/z 923.11

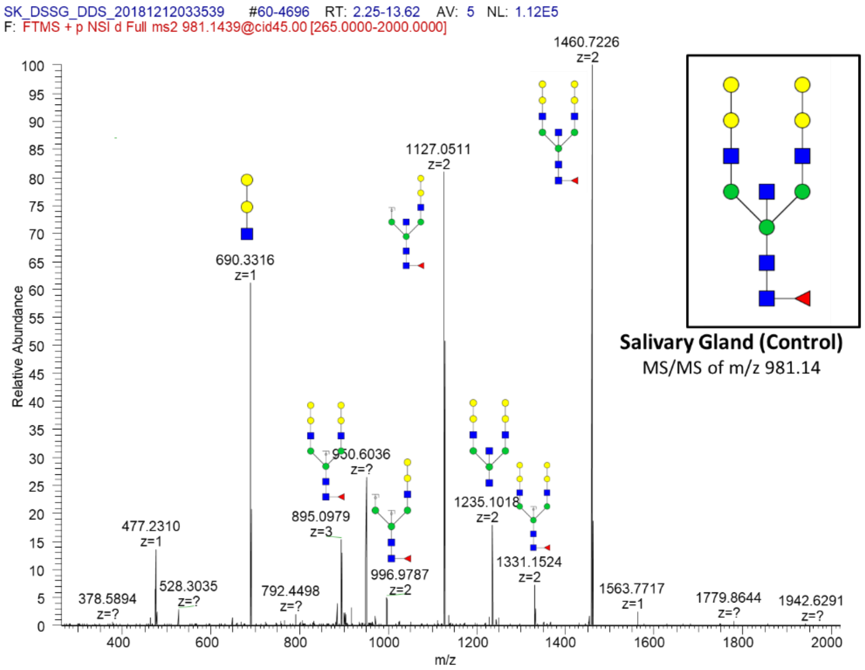

SK_DSSG_DDS_20181212033539 #60-4696 RT: 2.25-13.62 AV: 5 NL: 1.12E5
F: FTMS + p NSI d Full ms2 981.1439@cid45.00 [265.0000-2000.0000]
Relative Abundance
m/z
1460.7226 z=2
1127.0511 z=2
690.3316 z=1
950.6036 z=?
895.0979 z=3
1235.1018 z=2
477.2310 z=1
996.9787 z=2
1331.1524 z=2
528.3035 z=?
378.5894 z=?
792.4498 z=?
1563.7717 z=1
1779.8644 z=?
1942.6291 z=?
Salivary Gland (Control)
MS/MS of m/z 981.14

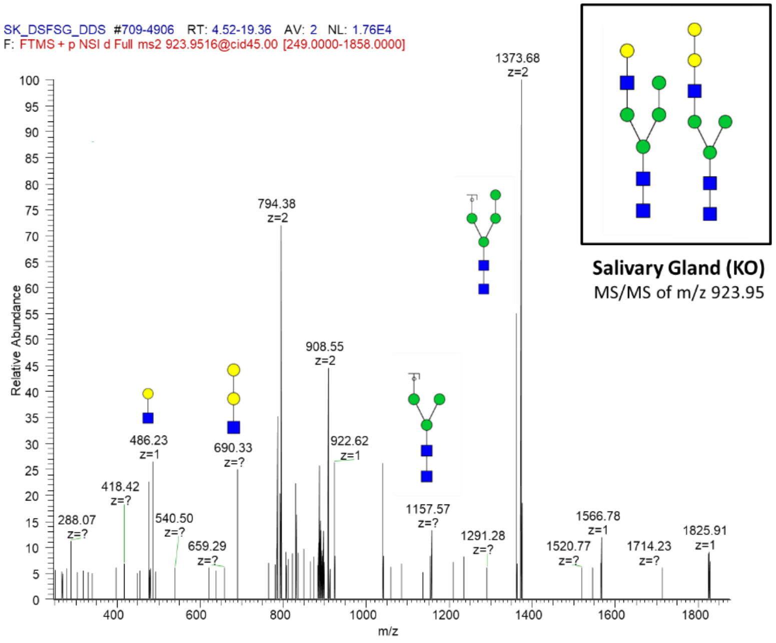

Salivary Gland (KO)
MS/MS of m/z 923.95

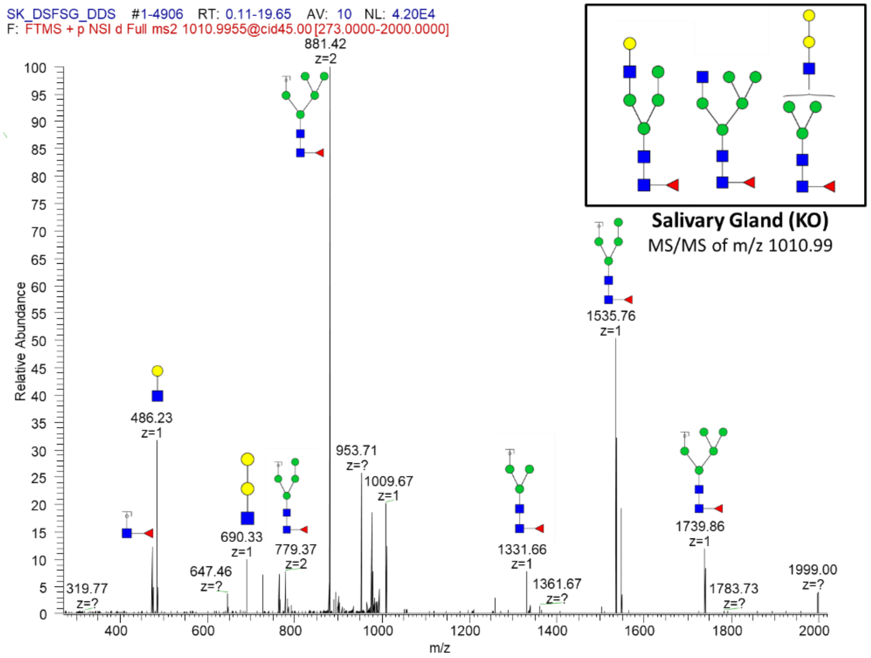

SK_DSFSG_DDS #1-4906 RT: 0.11-19.65 AV: 10 NL: 4.20E4
F: FTMS + p NSI d Full ms2 1010.9955@cid45.00 [273.0000-2000.0000]
Relative Abundance
m/z
881.42 z=2
486.23 z=1
690.33 z=1
779.37 z=2
953.71 z=?
1009.67 z=1
1331.66 z=1
1361.67 z=?
1535.76 z=1
1739.86 z=1
1783.73 z=?
1999.00 z=?
319.77 z=?
647.46 z=?
Salivary Gland (KO)
MS/MS of m/z 1010.99

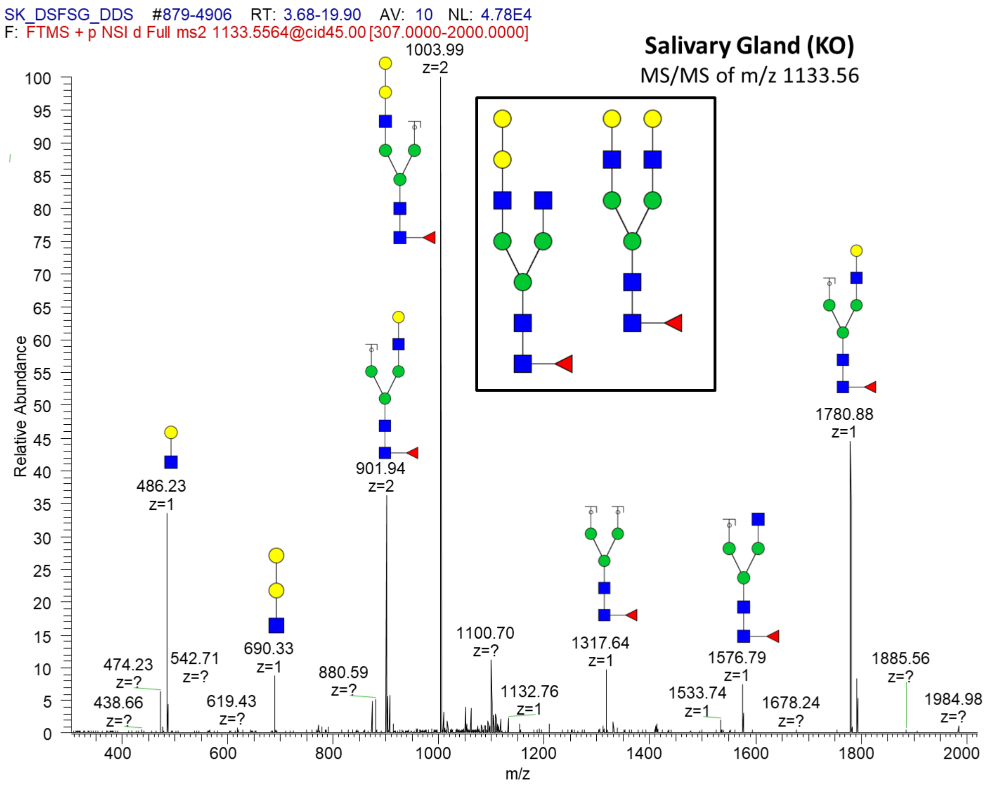

SK_DSFSG_DDS #879-4906 RT: 3.68-19.90 AV: 10 NL: 4.78E4
F: FTMS + p NSI d Full ms2 1133.5564@cid45.00 [307.0000-2000.0000]
Salivary Gland (KO)
MS/MS of m/z 1133.56
Relative Abundance
m/z
1003.99 z=2
901.94 z=2
486.23 z=1
690.33 z=1
1780.88 z=1
1317.64 z=1
1576.79 z=1
1100.70 z=?
1132.76 z=1
1533.74 z=1
1678.24 z=?
1885.56 z=?
1984.98 z=?
474.23 z=?
438.66 z=?
542.71 z=?
619.43 z=?
880.59 z=?

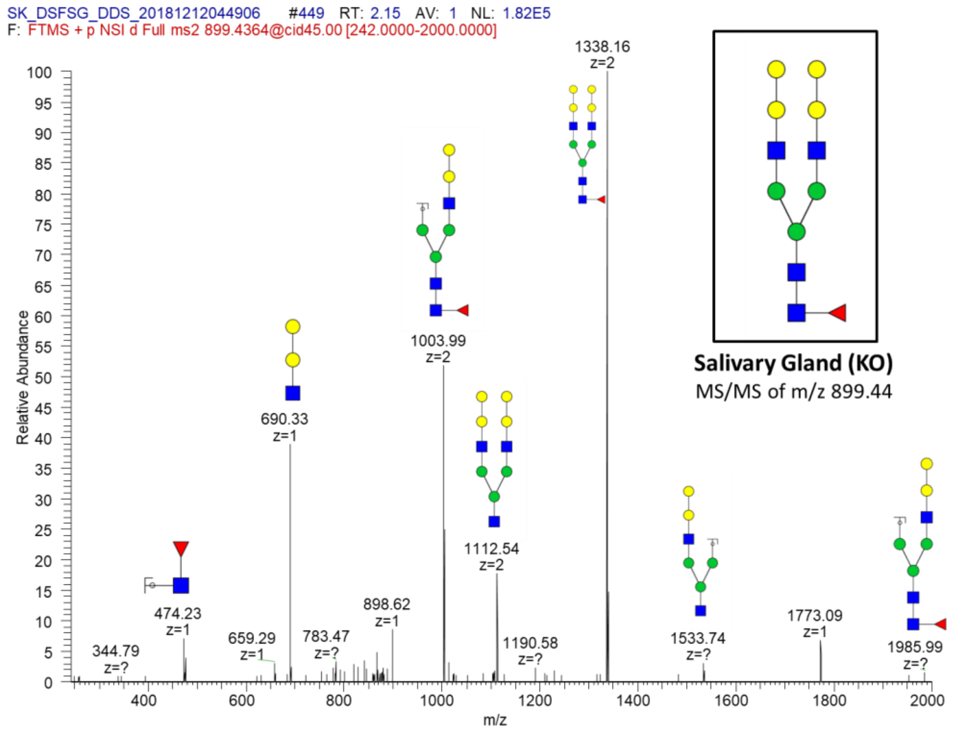
SK_DSFSG_DDS_20181212044906 #449 RT: 2.15 AV: 1 NL: 1.82E5
F: FTMS + p NSI d Full ms2 899.4364@cid45.00 [242.0000-2000.0000]
Relative Abundance
m/z
1338.16 z=2
1003.99 z=2
690.33 z=1
1112.54 z=2
474.23 z=1
898.62 z=1
659.29 z=1
783.47 z=?
1190.58 z=?
344.79 z=?
1533.74 z=?
1773.09 z=1
1985.99 z=?
Salivary Gland (KO)
MS/MS of m/z 899.44
