# Supplementary material for: Alpha-Gal Syndrome: Involvement of Amblyomma americanum α-D-Galactosidase and β-1,4 Galactosyltransferase Enzymes in α-Gal Metabolism
Source: Front Cell Infect Microbiol. 2021 Dec 1;11:775371. doi: 10.3389/fcimb.2021.775371 (PMC8671611; doi:10.3389/fcimb.2021.775371)
Supplement: Supplementary Table 1 — Summary of potential α-Gal Glycoforms Determined by MS/MS Analysis. [file Table_1.pdf]

Supplementary information

Supplementary Table 1: Summary of potential α-Gal Glycoforms Determined by MS/MS Analysis

	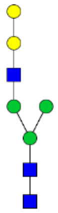	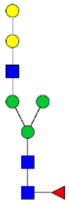	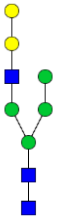	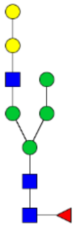	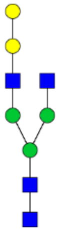	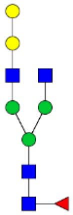	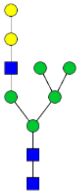	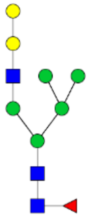	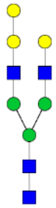	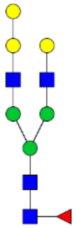
m/z	**1825**	**1999**	**2029**	**2203**	**2070**	**2244**	**2233**	**2407**	**2274**	**2448**
**SG-Control**		MIX			NO	MIX				
**SG-KO**	MIX	MIX			NO	MIX				

	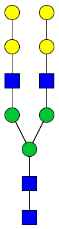	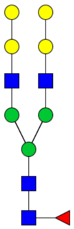	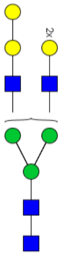	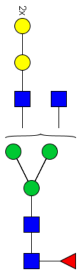
m/z	**2478**	**2652**	**2723**	**2897**
**SG-Control**	YES	YES	YES	YES
**SG-KO**		YES		YES

Greyed out boxes indicate that this mass was not detected in that sample.
“Unknown” = the MS/MS fragmentation was ambiguous,
“NO” = MS/MS fragmentation resulted in 486.23 ion
“YES” = MS/MS fragmentation resulted in 690.33 ion
YES/MIX” = MS/MS fragmentation resulted in both ions

Supplementary Table 2: List of genes, accession numbers, primers, and base sizes used in this study for transcriptional and gene silencing experiments.

**Gene**	**Accession**	**Forward primer 5’-3'**	**Reverse primer 5’-3'**	**Size (bp)**
Aa -Actin	EZ000248.1	TGGTATCCTCACCCTGAAGTA	ACGCAGCTCGTTGTAGAAG	100
Aa β-Tubulin	GBZX01001418.1	CACAGAAGCAGAGTCCAACA	CCTCCTCTTCATCTCCAAACTC	101
Aa Histone H3	GI:759084459	GAAGCCAGTGAGGCATACTT	GCTGGATATCCTTTGGCATGA	104
AamerSigP-37433 α-D-galactosidase	N/A	TCCGAACGACAACGAACTC	CTTGTGAATGTAGTCCGCTAGG	93
Aam-23951 β-1,4-N-acetylgalactosaminyl transferase	N/A	TCCAGTGCTTCGTGTTCC	TTTCTCGTGACGGACATGTG	100
Aam SigP-33934 STT3A	N/A	AGACTCTATTCTTTGGGGCAGTGACT	GCAAGTCAAAGAAGAAGGAGAACCACG	207
Aam-4310 galactokinase	N/A	GCAAGAACACGAAACACCTG	CAAATGTCCTTGAACTGCCAC	97
Aam41143 GALTT, uridyl (galactosyl transferase)	NA	AAAGATGAATGGGTCCTCGTATC	CACTTCAGACTGGCTCATCAA	100
T7 AamerSigP-37433 αD-galactosidase	NA	GTAATACGACTCACTATAGGGAGTTGGT CTGTTTCTTGCTTTTC	GTAATACGACTCACTATAGGGTACC CATCTTCAACGAGGTGATCT	193
T7 Aam-23951 β-1,4-N-acetylgalactosaminyltransferase	NA	GTAATACGACTCACTATAGGGGAGTCA GTGCCGTGAGTAAGGAG	GTAATACGACTCACTATAGGG TTCACTCGGCTTGCTCTTGGC	188
T7-GFP	NA	GAATTAATACGACTCACTATAGGGAGA GTCTTGTAGTTCCCGTCATCTT	GAATTAATACGACTCACTATA GGGAGAAGCCAACACTTGTCACTACTT	208

Supplementary table 3: FTMS of N-Glycans observed in control salivary glands.

$M+Na^{+}$	Δmass (Da)	Composition	Proposed Structure	Percentage (rel % intensity)
1345.6702	-0	$(Hex)_3$ $(HexNAc)_2$ $(Deoxyhexose)_1$	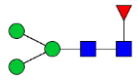	1.27%
1579.7874	0.004	$(Hex)_2$ + $(Man)_3(GlcNAc)_2$	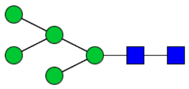	14.07%
1590.8036	0.004	$(HexNAc)_1$ $(Deoxyhexose)_1$ + $(Man)_3(GlcNAc)_2$	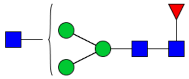	4.78%
1753.8774	0.005	$(Hex)_2$ $(Deoxyhexose)_1$ + $(Man)_3(GlcNAc)_2$	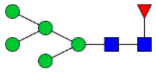	7.03%
1783.8878	0.005	$(Hex)_3$ + $(Man)_3(GlcNAc)_2$	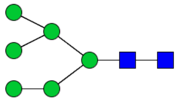	12.13%
1794.9002	0.002	$(Hex)_1$ $(HexNAc)_1$ $(Deoxyhexose)_1$ + $(Man)_3(GlcNAc)_2$	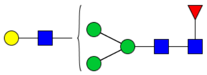	0.00%
1987.9888	0.006	$(Hex)_4$ + $(Man)_3(GlcNAc)_2$	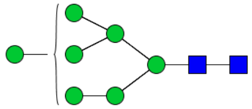	7.73%

1999.0046	0.006	(Hex)$_2$ (HexNAc)$_1$ (Deoxyhexose)$_1$ + (Man)$_3$(GlcNAc)$_2$	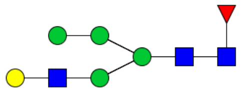 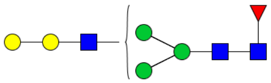	0.25%
2040.0254	0	(Hex)$_1$ (HexNAc)$_2$ (Deoxyhexose)$_1$ + (Man)$_3$(GlcNAc)$_2$	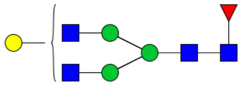	0.00%
2067.0416	0.005	(HexNAc)$_3$ (Pent)$_1$ + (Man)$_3$(GlcNAc)$_2$	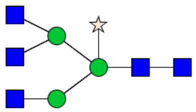	1.89%
2070.0444	0.009	(Hex)$_2$ (HexNAc)$_2$ + (Man)$_3$(GlcNAc)$_2$	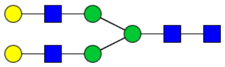	0.28%
2081.0572	0.006	(HexNAc)$_3$ (Deoxyhexose)$_1$ + (Man)$_3$(GlcNAc)$_2$	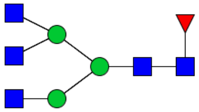	6.91%
2192.0876	0.005	(Hex)$_5$ + (Man)$_3$(GlcNAc)$_2$	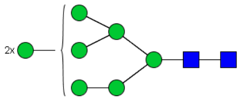	8.27%
2244.1248	0	(Hex)$_2$ (HexNAc)$_2$ (Deoxyhexose)$_1$ + (Man)$_3$(GlcNAc)$_2$	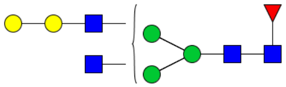 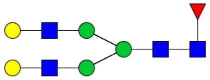	0.00%

2271.1448	0.009	$(Hex)_1$ $(HexNAc)_3$ $(Pent)_1$ + $(Man)_3(GlcNAc)_2$	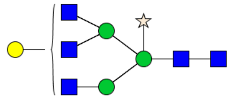	0.87%
2285.1566	0.005	$(Hex)_1$ $(HexNAc)_3$ $(Deoxyhexose)_1$ + $(Man)_3(GlcNAc)_2$	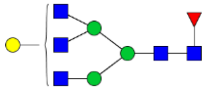	4.59%
2396.1884	0.006	$(Hex)_6$ + $(Man)_3(GlcNAc)_2$	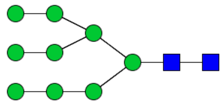	4.49%
2478.245	0.009	$(Hex)_4$ $(HexNAc)_2$ + $(Man)_3(GlcNAc)_2$	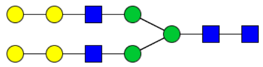	2.10%
2489.2474	-0	$(Hex)_2$ $(HexNAc)_3$ $(Deoxyhexose)_1$ + $(Man)_3(GlcNAc)_2$	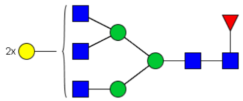	1.39%
2600.289	0.007	$(Hex)_7$ + $(Man)_3(GlcNAc)_2$	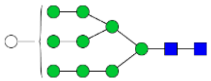	0.25%
2652.3286	0.004	$(Hex)_4$ $(HexNAc)_2$ $(Deoxyhexose)_1$ + $(Man)_3(GlcNAc)_2$	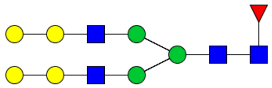	8.94%
2693.3543	0.003	$(Hex)_3$ $(HexNAc)_3$ $(Deoxyhexose)_1$ + $(Man)_3(GlcNAc)_2$	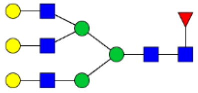	0.00%
2723.3663	0.005	$(Hex)_4$ $(HexNAc)_3$ + $(Man)_3(GlcNAc)_2$	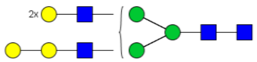 Multiple forms are possible for this structure	4.43%

2897.4492	0.013	(Hex)$_4$ (HexNAc)$_3$ (Deoxyhexose)$_1$ + (Man)$_3$(GlcNAc)$_2$	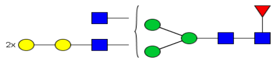 Multiple forms are possible for this structure	8.30%

Supplementary Table 4: FTMS of N-glycans observed in α-D-galactosidase silenced salivary glands

$M+Na^{+}$	Δmass (Da)	Composition	Proposed Structure	Percentage (rel % intensity)
1141.575	0.003	$(Hex)_2$ $(HexNAc)_2$ $(Deoxyhexose)_1$	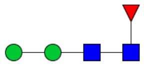	0.30%
1345.676	0.003	$(Hex)_3$ $(HexNAc)_2$ $(Deoxyhexose)_1$	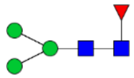	4.14%
1375.687	0.003	$(Hex)_4$ $(HexNAc)_2$	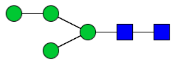	2.83%
1579.787	0.004	$(Hex)_2$ + $(Man)_3(GlcNAc)_2$	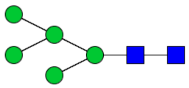	3.77%
1590.803	0.004	$(HexNAc)_1$ $(Deoxyhexose)_1$ + $(Man)_3(GlcNAc)_2$	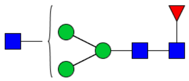	1.21%
1620.814	0.005	$(Hex)_1$ $(HexNAc)_1$ + $(Man)_3(GlcNAc)_2$	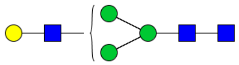	0.04%
1753.877	0.004	$(Hex)_2$ $(Deoxyhexose)_1$ + $(Man)_3(GlcNAc)_2$	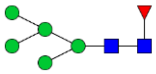	2.64%
1783.888	0.006	$(Hex)_3$ + $(Man)_3(GlcNAc)_2$	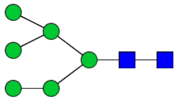	4.60%
1794.904	0.005	$(Hex)_1$ $(HexNAc)_1$ $(Deoxyhexose)_1$ + $(Man)_3(GlcNAc)_2$	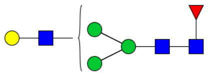	0.44%

1824.917	0.007	$(Hex)_2$ $(HexNAc)_1$ + $(Man)_3(GlcNAc)_2$	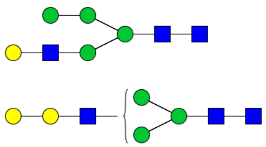	0.11%
1987.984	0.005	$(Hex)_4$ + $(Man)_3(GlcNAc)_2$	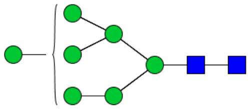	3.62%
1999.003	0.005	$(Hex)_2$ $(HexNAc)_1$ $(Deoxyhexose)_1$ + $(Man)_3(GlcNAc)_2$	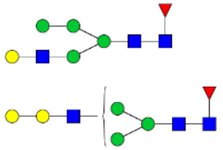	0.51%
2040.03	0.004	$(Hex)_1$ $(HexNAc)_2$ $(Deoxyhexose)_1$ + $(Man)_3(GlcNAc)_2$	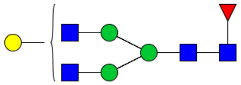	0.28%
2067.041	0.004	$(HexNAc)_3$ $(Pent)_1$ + $(Man)_3(GlcNAc)_2$	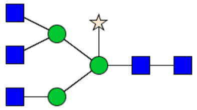	0.28%
2070.043	0.007	$(Hex)_2$ $(HexNAc)_2$ + $(Man)_3(GlcNAc)_2$	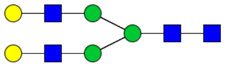	0.09%
2081.057	0.006	$(HexNAc)_3$ $(Deoxyhexose)_1$ + $(Man)_3(GlcNAc)_2$	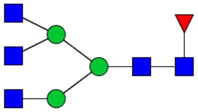	4.30%
2192.088	0.005	$(Hex)_5$ + $(Man)_3(GlcNAc)_2$	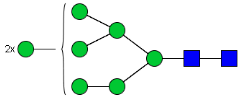	5.02%

2244.128	0.003	(Hex)$_2$ (HexNAc)$_2$ (Deoxyhexose)$_1$ + (Man)$_3$(GlcNAc)$_2$	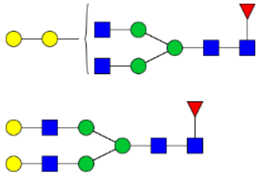	0.41%
2271.141	0.006	(Hex)$_1$ (HexNAc)$_3$ (Pent)$_1$ + (Man)$_3$(GlcNAc)$_2$	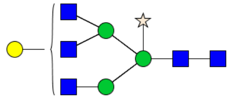	0.56%
2285.157	0.005	(Hex)$_1$ (HexNAc)$_3$ (Deoxyhexose)$_1$ + (Man)$_3$(GlcNAc)$_2$	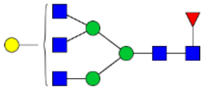	5.74%
2396.187	0.004	(Hex)$_6$ + (Man)$_3$(GlcNAc)$_2$	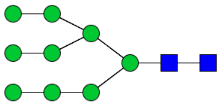	4.95%
2489.258	0.006	(Hex)$_2$ (HexNAc)$_3$ (Deoxyhexose)$_1$ + (Man)$_3$(GlcNAc)$_2$	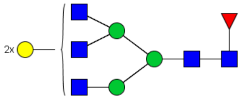	1.58%
2600.289	0.007	(Hex)$_7$ + (Man)$_3$(GlcNAc)$_2$	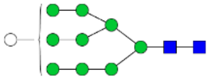	0.77%
2652.329	0.004	(Hex)$_4$ (HexNAc)$_2$ (Deoxyhexose)$_1$ + (Man)$_3$(GlcNAc)$_2$	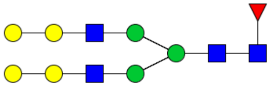	1.23%
2693.355	0.003	(Hex)$_3$ (HexNAc)$_3$ (Deoxyhexose)$_1$ + (Man)$_3$(GlcNAc)$_2$	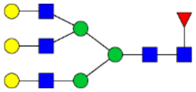	0.08%
2734.389	0.012	(Hex)$_2$ (HexNAc)$_4$ (Deoxyhexose)$_1$ + (Man)$_3$(GlcNAc)$_2$	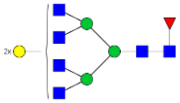	0.05%

2897.457	0.006	$(Hex)_4$ $(HexNAc)_3$ $(Deoxyhexose)_1$ + $(Man)_3(GlcNAc)_2$	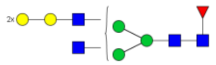 Multiple forms are possible for this structure	0.46%

Supplementary table 5: Frequency of Basophil activation

**Experiments**	**ADGal silenced (5dpi SG extracts)**	**No treatment (5dpi SG extracts)**
**Biological replicateR1 (N=5 tick)**	**9.14 %**	**13.3%**
**Biological replicateR2 (N=5 tick)**	**2.16%**	**9.51%**
**Mean**	**5.6%**	**11.4%**
